# Microplastics in Settled Dust from Indian Long-Distance Railway Coaches: Baseline Occurrence, Relative Load, and Scenario-Based Ingestion Estimates

**DOI:** 10.3390/toxics14090822

**Published:** 2026-09-15

**Authors:** Nisarg Mehta, Jing Wang, Barbara Kozielska

**Affiliations:** 1 Department of Air Protection, Faculty of Energy and Environmental Engineering, Silesian University of Technology, 22B Konarskiego St., 44-100 Gliwice, Poland; nisarg.dipakkumar.mehta@polsl.pl (N.M.); barbara.kozielska@polsl.pl (B.K.); 2 Empa, Laboratory for Building Energy Materials and Components, Überlandstrasse 129, 8600 Dübendorf, Switzerland; 3 Institute of Environmental Engineering, ETH Zürich, Laura-Hezner-Weg 7, 8093 Zürich, Switzerland

**Keywords:** microplastics in public transport, estimated daily intake, estimated annual intake, occupational health, indoor exposure, PCA, PET fibers

## Abstract

Microplastics (MPs) in long-distance trains remain undercharacterized despite prolonged exposure of passengers and staff in confined environments. In this study, settled dust was collected from four distinct coach classes on an Indian railway to establish baseline MPs occurrence, morphology, polymer composition, relative load, and exposure-relevant particle intake. Samples were processed via oxidative digestion, density separation, and stereomicroscopy for particle quantification and morphological characterization. Polymer identity was assessed using micro-Raman spectroscopy, and human exposure was evaluated using estimated trip (${EDI}_{Trip}$) and annual (EAI) intake models. MPs were detected in all four sampled coach composites, with coach-level concentrations ranging from 925 to 3755 MPs/g. Among the four sampled coaches, the 1AC (first-class sleeper air-conditioned) coach had the highest measured concentration. Fibers (72%), transparent particles (61%), and polyester dominated, suggesting mixed textile and interior sources. The Relative Load Ratio (RLR) reached 4.06 in 1AC, compared to the lowest-abundance general unreserved coach (GL). Deterministic ${EDI}_{Trip}$ ranged from 5.78 to 388.35 particles/trip, with maximum intake observed for toddlers on full-route 1AC journeys. Monte Carlo simulations confirmed intake increases with travel duration and frequency. These findings highlight long-distance railway coaches as overlooked MPs reservoirs, emphasize the need for improved dust control, ventilation management, and lower-shedding interior materials.

## 1. Introduction

Plastic pollution is a major and growing environmental problem, driven by increasing plastic production and inadequate management of plastic waste across its life cycle. Once released into the environment, larger items undergo mechanical abrasion, photo-oxidation, and chemical weathering, progressively fragmenting into microplastics (MPs), typically defined as synthetic polymer particles smaller than 5 mm in their longest dimension [1,2]. Initially, research and public concern focused on marine ecosystems, where floating and suspended plastic debris was first systematically reported [3]. However, it is now clear that MPs are ubiquitous across environmental contexts, including soils, freshwater systems, the atmosphere, and indoor environments [4]. This shift in focus has highlighted that humans are not only exposed via ingestion of contaminated food and water, but also via inhalation of airborne and resuspended particles in the built environment.

Indoor environments merit particular attention because people typically spend around 80–90% of their time indoors, and enclosed microenvironments can act as efficient sinks and secondary sources of MPs. A growing body of evidence demonstrates that indoor MPs originate from a combination of external inputs (e.g., tracked-in dust, outdoor air infiltration) and internal sources such as synthetic textiles, upholstery, floor coverings, household plastics, and technical systems [4,5]. Settled dust provides a useful matrix for characterizing MPs in indoor environments and for estimating human exposure through unintentional ingestion and resuspension of particles. Recent studies in homes, offices, schools, commercial settings, and hospitals have reported MPs abundances in indoor dust spanning two to three orders of magnitude and consistently dominated by polyester or polyethylene terephthalate (PET) polymers [6,7,8,9]. These findings suggest that indoor environments can sustain higher MPs concentrations than many outdoor settings and that human exposure in buildings may be non-negligible.

Despite this emerging evidence, key gaps remain in the understanding of MPs within specific indoor microenvironments and their implications for exposure. First, most work on inhalation exposure has focused on airborne MPs in apartments and offices, often using active air sampling and spectroscopy to quantify fibers and fragments in specific size ranges (e.g., 10–300 μm) [5,10]. Settled dust has been comparatively less investigated, even though dust resuspension is a well-established pathway for human contact with other indoor contaminants. Second, the distribution and determinants of MPs contamination and exposure in high-occupancy public or semi-public indoor spaces such as schools, hospitals, and transport systems are only beginning to be described. For example, studies in school classrooms and university facilities have shown that dust-bound MPs can reach hundreds to tens of thousands of particles per gram, with children potentially ingesting tens to hundreds of particles per day [6,7]. Third, MPs in indoor dust have recently been linked to inhalation exposure estimates in densely populated megacities such as Dhaka, where deposition fluxes and modeled daily doses suggest that inhalation can rival or exceed dietary intake under certain scenarios [8,11]. Nevertheless, quantitative exposure assessments remain sparse, and formal health-based benchmarks are not yet available [4,11].

Within this broader context, transport microenvironments remain understudied. People spend substantial, recurrent time in vehicles and transit systems, often under conditions of high occupancy, limited ventilation, and intensive use of polymer-rich interior materials. Recent studies on airborne MPs in car cabins and other transport modes indicate that concentrations can exceed those reported in many residential environments, and that polymer fingerprints often reflect standardized interior fittings, seat fabrics, and ventilation components [10,11]. Public transport environments, therefore, constitute an important but under-explored interface between indoor exposure science and urban sustainability.

Among public transport systems, long-distance passenger trains are particularly relevant in populous countries such as India, where railways serve as the primary mode of intercity travel for millions of passengers daily. These trains combine long journey durations, high passenger turnover, standardized interiors rich in synthetic textiles, and coach-specific ventilation regimes, creating socio-environmental conditions distinct from those in other indoor settings, such as homes, offices, or short-duration transit modes. Yet, empirical evidence of MPs contamination within train interiors remains extremely limited, and no systematic assessment has focused on settled indoor dust from long-distance passenger trains as an exposure-relevant matrix.

The present study addresses this gap by providing the first systematic assessment of MPs in settled indoor dust from a long-distance passenger train operated by Indian Railways, one of the world’s largest rail networks.

Accordingly, the primary objective of this research is to establish baseline information on the abundance, characteristics, and polymer composition of MPs in settled indoor dust across four individual coaches representing distinct microenvironments in a long-distance passenger train. This study’s specific objectives are (i) to quantify coach-specific MPs abundance and heterogeneity, (ii) to characterize dominant particle morphologies and micro-Raman-confirmed polymer signatures, and (iii) to estimate exposure-relevant particle intake using estimated trip intake (${EDI}_{Trip}$) and estimated annual intake (EAI), without assigning health-risk classifications because validated toxicological thresholds for environmental MPs are not yet available. Collectively, these objectives address a critical gap in the MPs literature by characterizing a previously understudied transport microenvironment and offering an empirical basis for future exposure-reduction and dust-control strategies.

MPs isolation, identification, and characterization, as well as the sampling strategy and protocol, were performed using the validated settled-dust workflow described in our recent studies [9,12,13,14], with modifications specific to the railway microenvironment summarized below. Detailed step-by-step analytical procedures, including oxidative digestion, density separation, vacuum filtration, stereomicroscopy, and micro-Raman spectroscopy, are provided in the Supplementary Information (Protocols S1 and S2).

### 1.1. Train Selection and Coach Classification

Settled dust was collected from an Indian Railways passenger train operating on the Rajkot Junction to Rewa Terminal route, Western Railway, India, a long-distance service covering 1601 km in approximately 27.6 h with 24 intermediate stops. To examine variability in MPs accumulation associated with coach enclosure, passenger use, and ventilation regime, a single composite sample was obtained from each of four individual coach categories, which are namely: general (GL, unreserved coach), sleeper (SL, reserved non-air-conditioned sleeper coach), three-tier sleeper air-conditioned coach (3AC), and first-class sleeper air-conditioned coach (1AC). Within each individual carriage, settled dust was collected from the flooring. This stratified design allowed direct comparison along a socioeconomic and environmental gradient within a single long-duration travel system.

### 1.2. Sampling Protocol

Settled dust was sampled during the winter season. At the time of sample collection, external ambient conditions were dry, with relative humidity ranging from 30% to 50% and temperature ranging from 15 °C to 28 °C. This seasonal window was selected to characterize MPs deposition during a period when reduced rainfall and lower humidity can favor dust accumulation and retention, while avoiding the washout effects associated with the monsoon season. Winter travel may also reduce natural ventilation in non-AC coaches because windows are more likely to remain closed for thermal comfort, potentially increasing particle retention within coach interiors. This approach is consistent with urban dust and MPs studies in India that evaluate particle accumulation under dry-season conditions [15,16].

Sampling was conducted within 1–2 h after journey completion during the scheduled routine-cleaning period in coordination with the housekeeping team during the initial dry-cleaning phase at the railway yard. Specifically, the sampling process consisted of manual sweeping. To ensure standardization, sample dust collection from all four coaches was conducted in the exact same manner, and dust was collected exclusively from the same functional areas, specifically the accessible PVC flooring across the passenger area of each coach (approximately 58–60 m^2^, as detailed in Table S1).

This post-journey timing was selected to capture dust accumulated over the full operational cycle, including contributions from passenger activity, textile abrasion, luggage movement, ventilation conditions, and interior wear under actual travel conditions. To minimize plastic contamination, natural-bristle brooms were used to collect a settled-dust sample from each of the four coach classes. The coach-specific metadata (e.g., ventilation status) are detailed in Supplementary Table S1.

A total of 5–10 g of settled dust from each coach was collected as a composite dust sample. The dust samples were transferred directly into pre-cleaned paper bags and transported to the laboratory under clean, dry conditions.

Before analysis, each composite sample was thoroughly homogenized and divided into analytical replicate subsamples (*n* = 3 per coach; total of 12) to evaluate measurement consistency and within-sample variability. This approach follows established indoor MPs studies using dust as an integrated exposure matrix [7,9,13,17].

### 1.3. Quality Assurance and Quality Control

To minimize secondary contamination, quality-assurance and quality-control measures were implemented throughout sampling, sample preparation, and analysis. During sampling, a separate, natural-bristle broom was dedicated to each coach. Only glass and metal equipment were used, personnel wore nitrile gloves and cotton laboratory clothing, and work surfaces and instruments were cleaned with ethanol before each procedure. Analytical replicates were used to check within-sample homogenization and analytical variability during the extraction workflow. Reagents were pre-filtered, and procedural blanks (*n* = 4; one blank per coach) were processed in parallel with the samples. No MPs were observed in procedural blanks above the stereomicroscopy detection limit (<20 μm). Micro-Raman spectra of blanks also showed no polymer signatures attributable to laboratory sources, indicating negligible background contamination. Extensive step-by-step QA/QC protocols, including analyst identification criteria and filtration pore sizes, are detailed in the Supplementary Information (Protocol S3).

In the present study, exposure-relevant particle intake was estimated through settled-dust ingestion using estimated trip intake (${EDI}_{Trip}$) and estimated annual intake (EAI). These estimates were calculated for different age groups, coach classes, travel duration scenarios, and annual travel frequency assumptions. The details are presented in the Supplementary Information (Protocol S4.1).

Monte Carlo simulation was used to propagate uncertainty in particle-intake estimates. Coach-specific MPs concentrations and dust ingestion rates were treated as uncertain inputs, while travel duration and annual travel frequency were treated as scenario variables. For each scenario, 20,000 iterations were performed, and outputs were summarized as P05, P50, and P95. Spearman’s rank correlation was used as an exploratory sensitivity indicator. Monte Carlo simulations were performed in R using *dplyr*, *tidyr*, *purrr*, and *readr* for data handling, scenario generation, iteration, and output export; detailed input parameters are provided in Tables S2–S4 in the Supplementary Information (Protocol S4.2).

### 1.4. Statistical and Compositional Analysis

An exploratory statistical framework was applied to evaluate relationships between MPs characteristics (color, shape, and size), their abundance, and coach-specific variability across train coaches. The overall MPs distribution was first assessed using location-wise total abundances and summarized with descriptive statistics (mean, SD, median, range, and coefficient of variation [CV]). The CV values were emphasized alongside descriptive and exploratory results to quantify spatial heterogeneity, with higher CVs indicating patchy or episodic contamination and lower values reflecting more uniform deposition, as commonly interpreted in previous indoor MPs studies [9,13].

To quantify coach-specific accumulation without relying on an artificial, uncontaminated background, a Relative Load Ratio (RLR) was calculated as the fold difference in MPs concentration relative to GL. GL was selected because it had the lowest measured MPs abundance in this sampling campaign and was not treated as a clean background or uncontaminated reference. Morphological diversity was described using Simpson’s diversity index for color, shape, and size only. The mathematical formulation is provided in the Supplementary Information (Protocol S5).

To visually explore the descriptive differences in MPs’ morphological composition among the four sampled coaches (SL, GL, 3AC, and 1AC), an exploratory multivariate compositional analysis was performed using particle-count data for shape, size class, and color. Raw counts were converted to row-wise percentages so that each variable represented its relative contribution within a sample. Because these data are compositional, percentages were transformed using the centered log-ratio (CLR) approach after adding a small pseudocount (e.g., 0.01%) to handle zero values. The exact input matrices are provided in Table S5.

Principal component analysis (PCA) [18] was then performed on the CLR-transformed dataset to summarize the major gradients of variation among samples and morphological classes. Hierarchical clustering and clustered heatmap visualization were subsequently conducted on the same CLR matrix using Euclidean distance and Ward’s minimum variance clustering method (ward.D2). A more detailed description of data preprocessing, CLR transformation, clustering procedure, and graphical settings is provided in the Supplementary Methods (Protocol S6).

All analyses and visualizations were performed in R using RStudio (version 2026.01.0, Build 392). Data processing and statistical analyses were conducted using “*dplyr*” (version 1.1.4) and other “*tidyverse*” packages for data wrangling, compositions for centered log-ratio (CLR) transformation, “*factoextra*” for PCA visualization, “*pheatmap*” for heatmaps, and “*ggplot2*” (version 3.5.2) for publication-quality figures.

## 2. Results and Discussion

### 2.1. Spatial Distribution of Microplastics in Railway Coaches

MPs were detected in all four sampled coach composites. The coach-level concentrations were 925 ± 681 MPs/g in GL, 1168 ± 856 MPs/g in 3AC, 1785 ± 425 MPs/g in SL, and 3755 ± 1487 MPs/g in 1AC (Table 1). The CV of 0.67 indicates substantial heterogeneity among coaches and falls within the range reported for other indoor dust MPs studies (CV = 0.50–1.20) [6,9].

**Figure 1:**
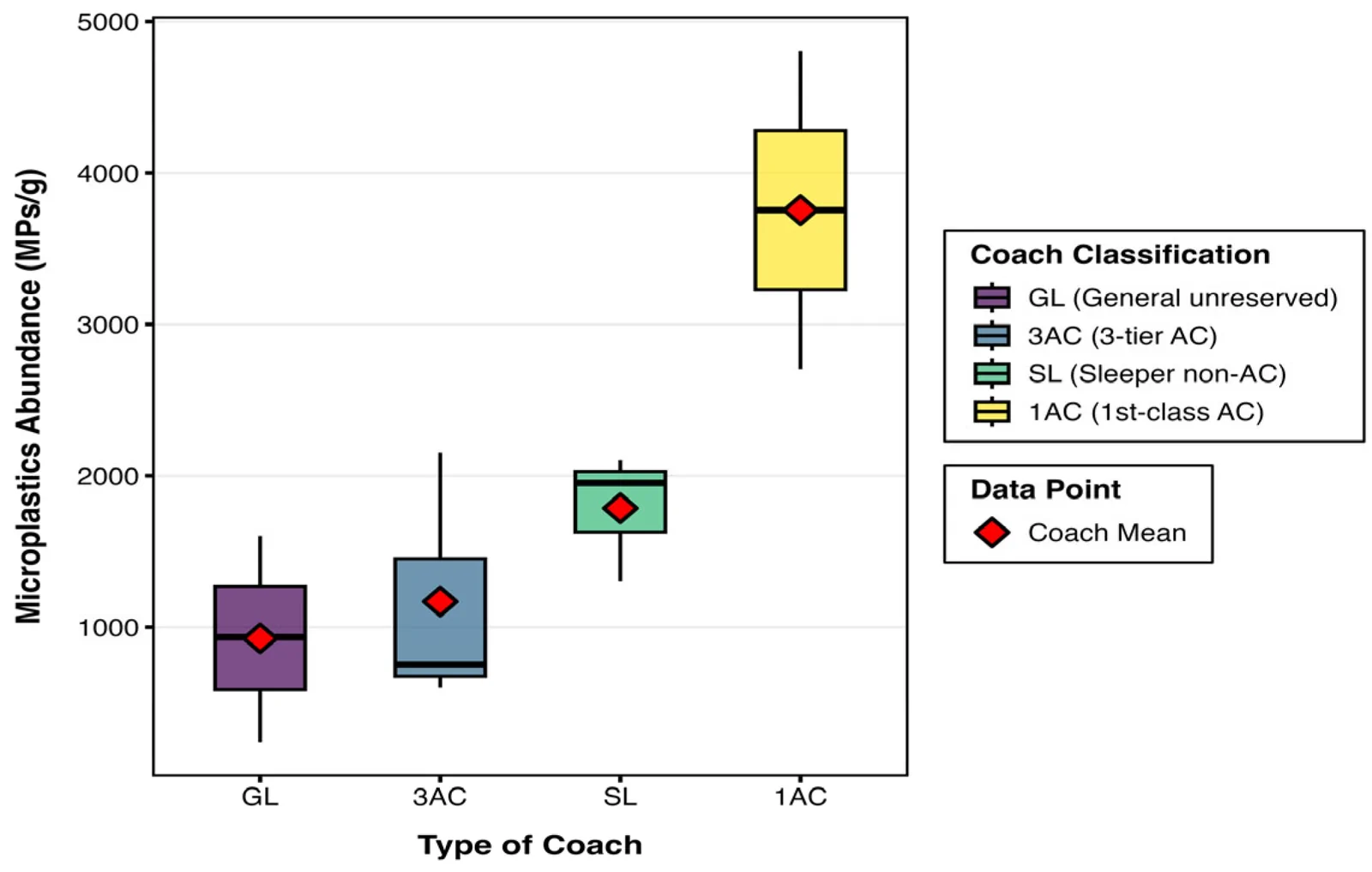
Distribution of microplastic concentrations (MPs/g) across sampled coaches (n=4 coach composites, each analyzed in triplicate; total N=12 analytical determinations). The median (center line), interquartile range (box), and mean (red diamond) reflect the overall train microenvironment distribution.

The differences observed among the sampled coaches (1AC > SL > 3AC > GL; Figure 1; Table S6) likely reflect coach-specific differences (Table S1) in MPs sources, ventilation, and deposition dynamics. Among the four coach composites sampled, the higher MP abundance observed in the 1AC coach must be interpreted through a combination of its sealed architectural design and its specific interior furnishings (Table S1). While the primary seating consists of low-shedding vinyl or artificial leatherette, the 1AC microenvironment uniquely incorporates extensive supplementary textiles, such as privacy curtains and provided passenger bedding. The mechanical abrasion of these materials, combined with continuous shedding from personal clothing and luggage, generates synthetic particles that become highly concentrated within the sealed, air-conditioned cabin (which features only 10 sealed windows). Consequently, even though the 1AC coach has the lowest passenger occupancy (24 passengers), the complete lack of natural ventilation prevents particle dilution, leading to maximum localized accumulation, a mechanism previously reported for MP retention in enclosed environments [19,20,21,22]. The relatively confined geometry and restricted air exchange associated with dedicated air-conditioning systems in the 1AC coach may further promote MPs accumulation, creating a localized retention effect [23].

In contrast, the lower MP abundance observed in the GL coach is likely driven by a combination of high air exchange rates and a lack of internal textile sources (Table S1). The GL coach features extensive natural ventilation through 36 open windows and six open doors, which facilitates continuous particle dilution and removal. Furthermore, the interior furnishings consist entirely of non-shedding materials (vinyl or artificial leatherette) with no curtains or provided bedding, fundamentally limiting the internal generation of synthetic fibers. Therefore, while the GL coach experiences the highest passenger occupancy (100–108 passengers per 58–60 m^2^)—which inherently introduces substantial shedding from personal clothing and luggage—this high continuous input is effectively offset by the aggressive natural ventilation and the absence of shed-prone architectural textiles. The intermediate values in SL and 3AC may be influenced by a balance of passenger density and partial enclosure. The relatively small difference between SL and GL concentrations (860 MPs/g) compared to the 1AC–GL gap (2829 MPs/g) reflects a qualitative distinction between well-enclosed and more open coach environments.

The observed variability (CV = 0.67) points to heterogeneous contamination within the train system, likely driven by a complex interplay of factors, including (1) distinct differences in natural versus sealed mechanical ventilation systems; (2) the varying presence of internal textile sources (e.g., privacy curtains and provided bedding in AC classes versus bare vinyl seating in unreserved classes), alongside continuous shedding from personal clothing and luggage; and (3) pronounced variations in passenger density (ranging from 24 to over 100 occupants) and spatial occupancy dynamics.

These findings add to the limited data on MPs abundance in public transportation settings, where data remain sparse compared to other indoor environments. The mean concentration of 1908 MPs/g exceeds levels reported in university classrooms (209 MPs/g) and dormitories (775 MPs/g) by Zhu et al. [7], as well as school dust in Iran (195 MPs/g) by Nematollahi et al. [6], but is lower than those in industrial and commercial indoor dust in Dhaka (6081 ± 396.3 MPs/g), with an overall mean across all indoor environments of 4333.18 ± 353.85 MPs/g by Haque et al. [8] and comparable to university laboratory dust in Colombia (1635 MPs/g) by Avilés Valera et al. [24]. Notably, a recent study by Zhai et al. [25] at a Chinese university indoor environment documented MPs abundances ranging from 632 to 3424 MPs/g across different locations (laboratories, classrooms, conference rooms), with significant between-locations variation attributed to differences in human activity intensity, textile usage, and mechanical ventilation efficiency. Relative to these international benchmarks, the present concentrations are moderate to elevated for enclosed public spaces.

These coach-specific patterns identify long-distance public transit as a critical, underexamined microenvironment for MPs accumulation. Crucially, they demonstrate that MP deposition within these train systems is governed by a highly dynamic interplay of variables. Rather than being driven by a single architectural parameter, the localized MPs load is collectively shaped by the specific configuration of ventilation systems (sealed versus natural), the presence of shedding-prone interior textiles (such as curtains and provided bedding), and human behavioral factors, including extreme variations in occupant density and continuous shedding from personal clothing and luggage.

### 2.2. Microplastics Morphology in Railway Coaches

#### 2.2.1. Microplastics Color Distribution

**Figure 2:**
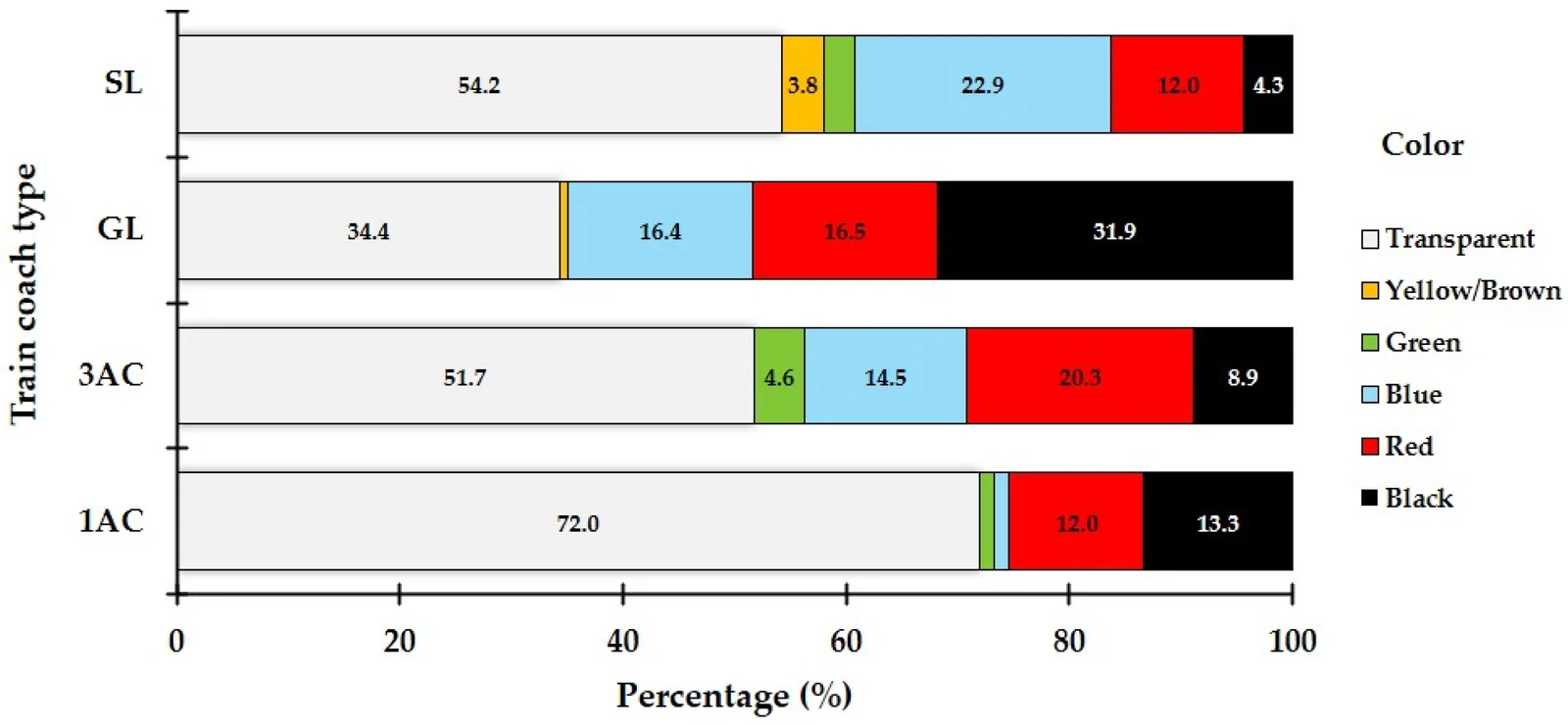
Microplastics color composition in train coach dust (SL, GL, 3AC, 1AC). Bars show the percentage (%) of each color category within the four coach composites (n=4, one composite per coach); analytical subsamples were combined at the coach level.

Color composition varied descriptively among the individual sampled coaches. Transparent particles were dominant across coaches, comprising 72.0% in 1AC, 51.7% in 3AC, 54.2% in SL, and 34.4% in GL (Figure 2). This aligns with indoor dust studies showing that transparent MPs, often 30–60% of total counts, may derive from clear packaging films and colorless synthetic fibers in high-occupancy spaces [8,24]. Linked to polyethylene terephthalate (PET) and polyesters, these MPs likely reflect textile degradation under mechanical stress [5,26]. In 1AC, dominance by transparent MPs, alongside fiber-rich morphology (see Section 2.2.2), is consistent with potential shedding from a combination of sources, such as upholstery, curtains, and clothing in enclosed settings [24,27,28]. The transparent, fiber-rich profile in first-class coaches likely reflects textile abrasion as the possible source, combined with relatively low airflow disturbance that promotes settling and retention of released fibers over time. Identification of PET in our samples strengthens this link, though limited spectroscopic analysis risks missing other polymers below detection limits. The polymer evidence should be viewed as supportive rather than exhaustive [8,26].

In contrast, black MPs were most abundant in GL coaches. These coaches typically experience high passenger density and frequent luggage handling, conditions that favor abrasion and resuspension processes. Black particles in indoor dust can originate from black rubber or technical plastics in luggage wheels, footwear soles, coach fittings, and black textiles or dark clothing fibers. Tire- and road-wear dust tracked into coaches through open windows and doors is a plausible additional source of black particles in GL; the specific contribution of this outdoor-to-indoor pathway was not directly quantified in this study.

Blue MPs were preferentially associated with non-air-conditioned coaches, being overrepresented in SL and GL but underrepresented in 1AC. This distribution likely reflects a greater contribution from colored synthetic fibers and fragments derived from passenger clothing, luggage, and interior textiles, such as seat fabrics and curtains [27].

In Indian Railways, SL, GL, and 3AC coaches predominantly feature blue seat fabrics and curtains, whereas 1AC interiors are largely red-toned (Figure S1), providing a route-specific explanation for elevated blue MPs in non-AC coaches. Blue particles are also indicative of textile abrasion from passenger clothing in high-occupancy settings, driven by movement-induced fabric friction [29].

Red MPs accounted for approximately 12–20% of particles in all coaches. This distribution suggests mixed sources, including dyed textiles and red packaging or labeling materials associated with luggage and freight [30]. In absolute terms, red MPs were most numerous in 1AC (452 particles; Table S6), suggesting a substantial local contribution from the red interior furnishings of this coach (Figure S1). This interpretation is consistent with evidence that indoor MPs are frequently generated by abrasion and shedding of textiles, upholstery, and furniture surfaces, which release colored fibers and fragments into dust. Thus, red seat and curtain materials in 1AC likely act as a possible source of red MPs under repeated passenger use and fabric friction. Minor color groups (yellow/brown and green), though comprising small fractions, displayed non-random distributions indicative of contributions from passenger belongings and external dust rather than the dominant sources [4].

These findings point to a role for coach-specific microenvironments in shaping color composition and support targeted mitigation strategies, such as improved filtration in air-conditioned coaches and material upgrades in high-traffic coaches. Longitudinal sampling will be essential to resolve temporal variability and refine source attribution.

#### 2.2.2. Microplastics Shape Distribution

**Figure 3:**
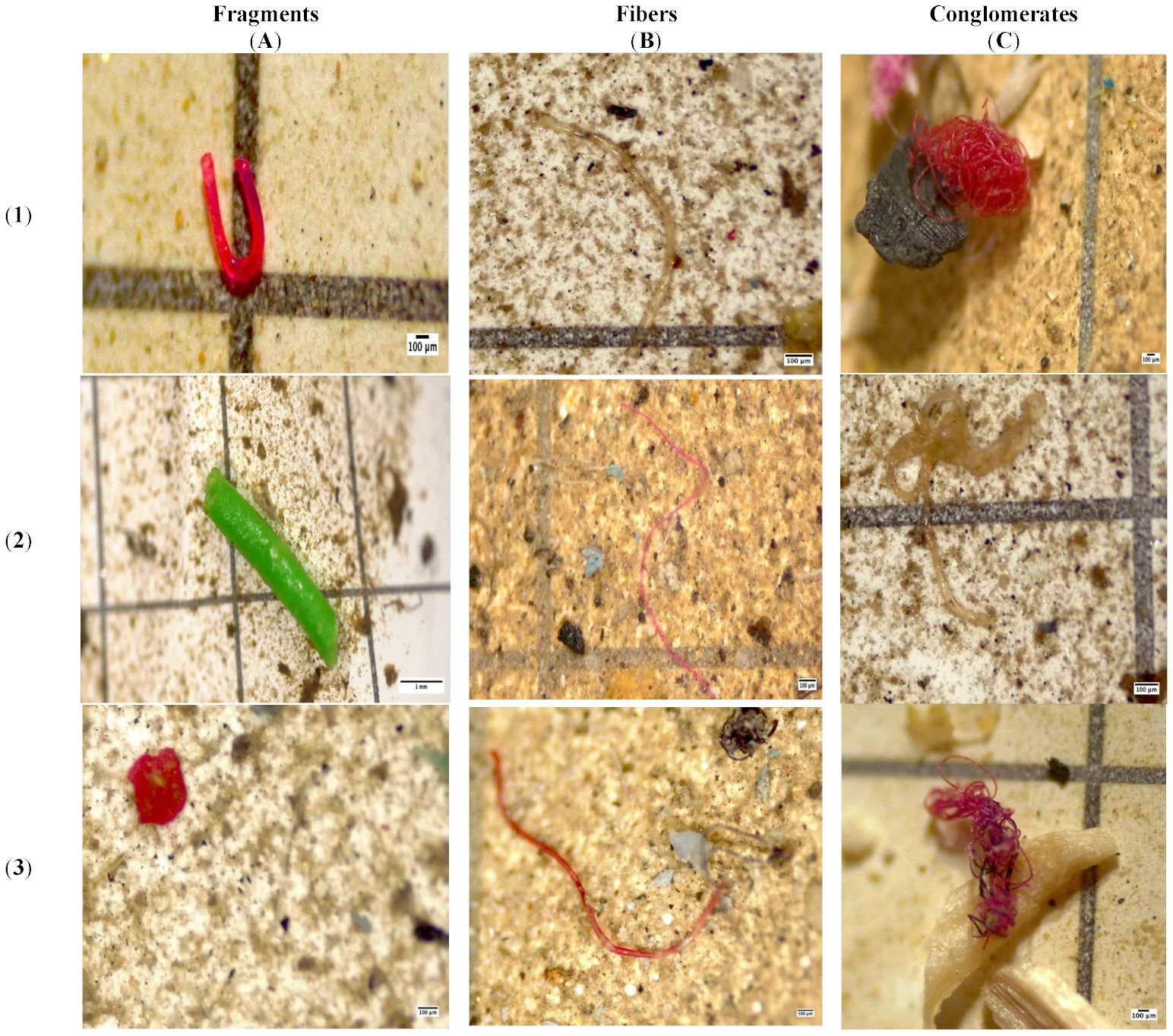
Representative optical microscopy images of microplastic fibers, fragments, and conglomerates identified in settled dust from four train coach composites (*n* = 4). Panels are arranged by row (**1**–**3**) and column (**A**–**C**): (**1A**) red fragment from GL; (**1B**) transparent fiber from 1AC; (**1C**) red conglomerate from GL; (**2A**) green fragment from 3AC; (**2B**) red fiber from GL; (**2C**) transparent conglomerate from 3AC; (**3A**) red fragment from SL; (**3B**) red fiber from SL; and (**3C**) conglomerate from GL. Scale bars are shown on individual micrographs.

The physical morphology of MPs provides insight into emission pathways, transport behavior, and potential exposure relevance. Dense, intertwined fiber aggregates of single or mixed colors were frequently observed; because individual fibers within these clusters could not be reliably enumerated, they were classified as conglomerates and counted as single units, consistent with established indoor MPs assessment protocols. Three primary morphologies were identified: fibers, fragments, and conglomerates (Figure 3).

**Figure 4:**
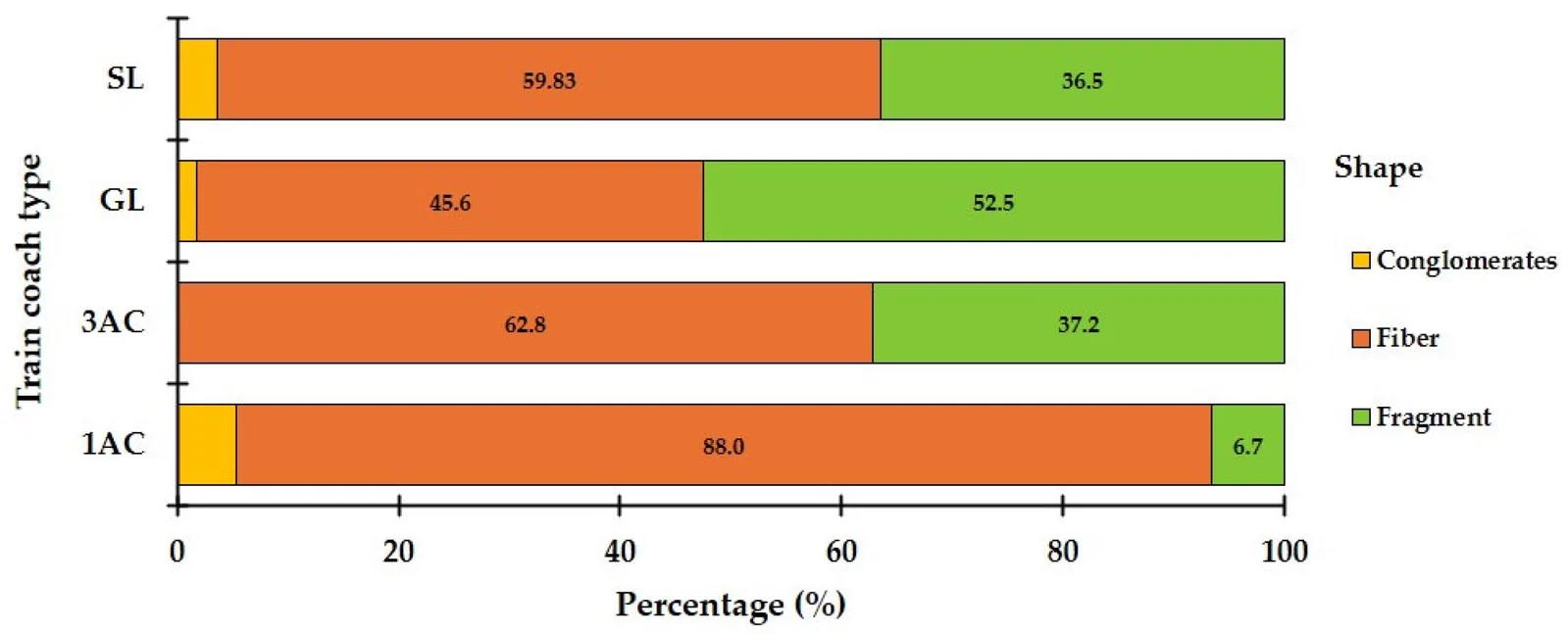
Morphological classification of microplastics in settled dust from railway coach dust. The Stacked bar chart shows the relative abundance of microplastic shapes (e.g., fibers, fragments, conglomerates) within four coach composites (n=4, one composite per coach).

Overall, across the train environment, fibers constituted the predominant morphological class, followed by fragments, while conglomerates represented a comparatively minor fraction of the total MPs detected (Figure 4). This distribution pattern is characteristic of indoor and semi-enclosed microenvironments and reflects the dominance of textile-derived emissions in spaces with sustained human occupancy. Similar fiber dominance has been reported in a wide range of indoor and transport-related studies, including subway systems, offices, and residential dust, where fibers commonly account for 50–90% of total MPs due to continuous shedding from clothing, upholstery, and synthetic furnishings [5,10,31,32].

The 1AC fiber load of 88% exceeds typical indoor baselines, consistent with sustained microfiber release under low-turbulence conditions and PET detection as the sole identified polymer [26]. Conglomerate enrichment may reflect fiber aggregation onto sticky surfaces or dust trapping by soft furnishings, a pattern noted in upholstered environments [33]. This enrichment is consistent with the enclosed nature of 1AC coaches and limited air exchange, which collectively promote the accumulation and retention of low-density fibrous particles. Previous studies have similarly identified air-conditioned indoor environments as preferential sinks for fibrous MPs due to reduced turbulence and efficient trapping within ventilation systems and filters [10].

Conversely, fragment dominance (36–53% across SL/GL/3AC) may reflect mechanical degradation of rigid plastics—luggage fittings, packaging, and interior components—plus resuspension of brittle debris in high-occupancy coaches [30,34,35]. Fragment-rich profiles mirror transport studies, such as 70% fragments in Istanbul subways and elevated breakage products in Korean rail systems [32,35].

Conglomerates were more prevalent in 1AC coaches, suggesting aggregation of fibers and fine particles under low-turbulence, enclosed conditions. Such aggregation phenomena have been increasingly recognized in indoor MPs studies, where prolonged residence times and limited air circulation facilitate particle–particle interactions and cluster formation [36].

Collectively, these findings indicate a morphological difference between air-conditioned and non-air-conditioned train coaches: fibrous MPs accumulate preferentially in enclosed environments, while fragments dominate in more open coaches subject to greater mechanical disturbance and external inputs. The enrichment of fibers in premium, enclosed coaches is relevant to passenger exposure and points to a need for targeted mitigation strategies focusing on textile materials and ventilation management.

#### 2.2.3. Microplastics Size Distribution

**Figure 5:**
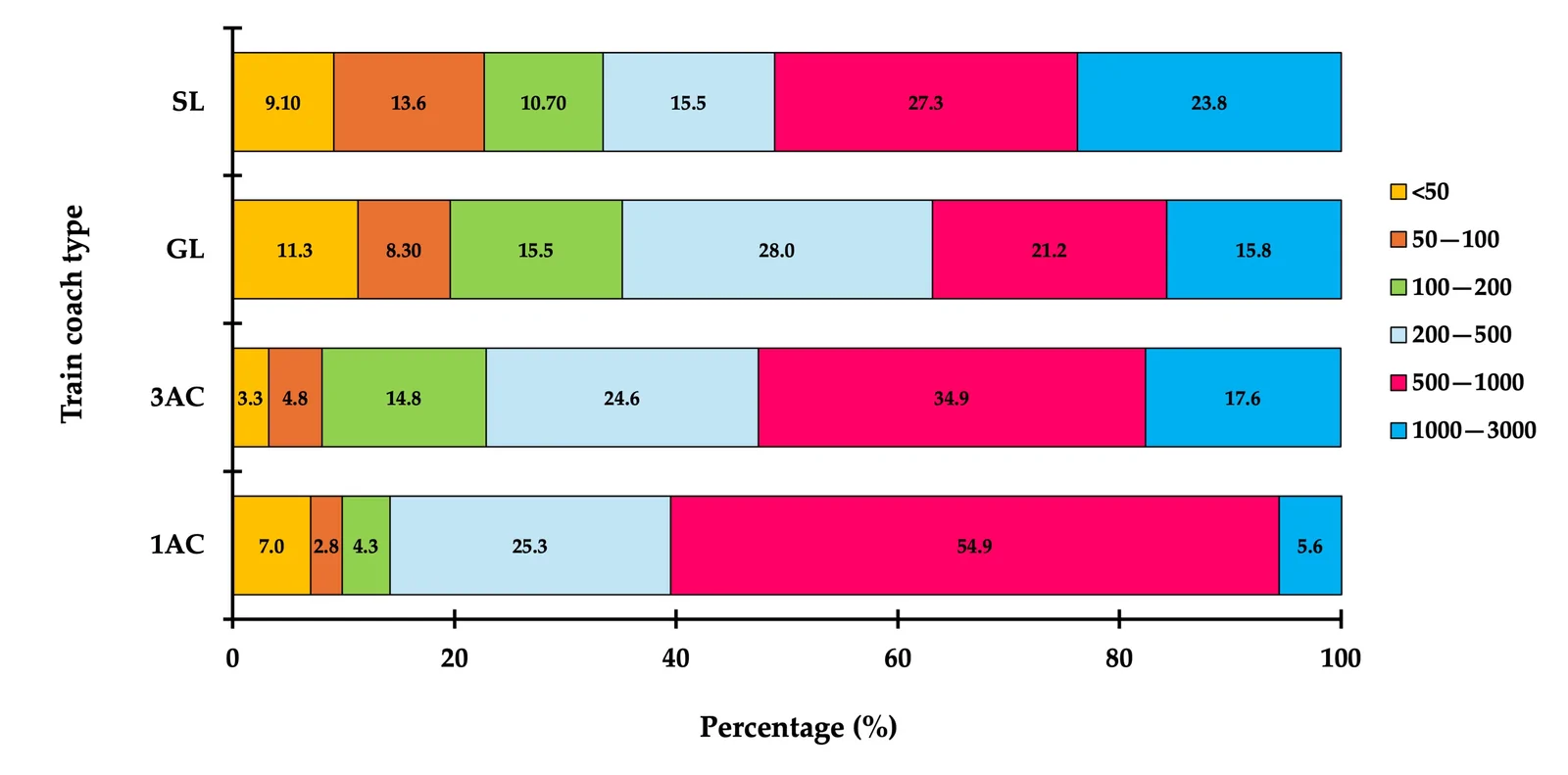
Microplastics size distribution by coach type. Percentage composition of MPs size classes (µm) in SL, GL, 3AC, and 1AC coaches (n=4, one composite per coach); analytical subsamples were combined at the coach level.

Size composition showed a clear descriptive contrast among the sampled coaches. Among the MPs visually detected and classified by optical microscopy, the 500–1000 µm fraction dominated overall (Figure 5), accounting for 27.28% in SL, 21.15% in GL, 34.93% in 3AC, and 54.91% in 1AC. This mid-to-large-size dominance aligns with indoor studies reporting that 100–1000 µm particles comprise 30–60% of MPs in occupied buildings, reflecting balanced degradation and gravitational settling in enclosed spaces [37,38,39].

Fine-particle enrichment (<200 µm) in SL/GL implicates advanced fragmentation and resuspension enhanced by high occupancy, luggage movement, and turbulent airflow, processes that may increase smaller MPs fractions in crowded transport microenvironments [21,32]. The 1AC’s coarse-particle dominance (500–1000 µm: 54.91%) may reflect retention of larger particles and/or removal, redistribution, or under-recovery of finer fractions [33]. It is also possible that part of the fine fiber fraction agglomerated into larger conglomerates in this coach, thereby increasing the apparent coarse-size contribution. This size–shape pattern of large fibers/conglomerates in 1AC versus fine fragments in SL/GL is consistent with activity-specific emission pathways [30].

The SL/GL fine-particle bias parallels elevated <100 µm fractions in high-traffic subways [35], while 1AC’s coarse profile resembles low-turbulence environments retaining larger fragments [39]. Size structuring also challenges uniform exposure models: 1AC exhibited the highest overall MPs abundance but was dominated by coarser particles, while SL/GL contained relatively finer fractions, indicating different exposure profiles rather than lower versus higher total loads [40].

### 2.3. Multivariate Analysis of Microplastic Morphology

**Figure 6:**
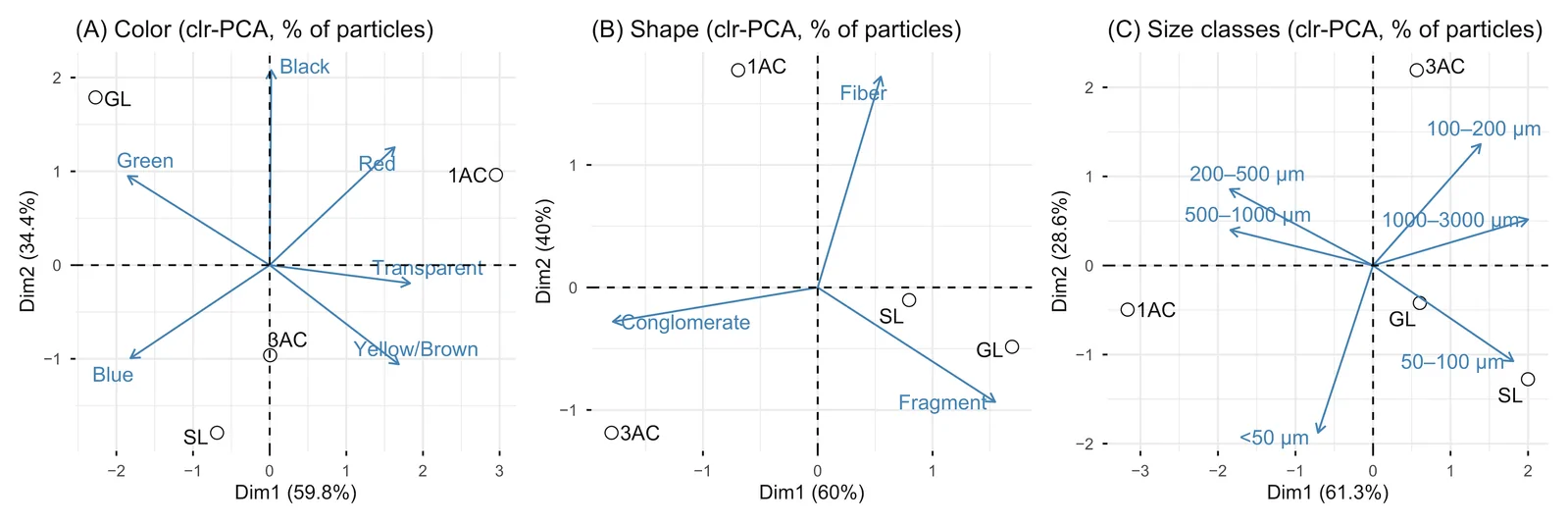
Exploratory CLR-PCA of microplastic composition in four train coach classes. (**A**) Color composition showing the separation of SL, GL, 3AC, and 1AC along gradients from transparent to dark particles. (**B**) Shape composition showing contrasts among fragment-dominated, fiber-rich, and conglomerate-rich profiles. (**C**) Size-class composition highlighting enrichment in large (200–3000 µm) versus fine (<100 µm) particles. All biplots are based on CLR-transformed percentages, and the first two PCs explained 94.2%, 100.0%, and 89.9% of the variance in the color, shape, and size PCAs, respectively.Open circles represent the four coach composites (SL, GL, 3AC, and 1AC; *n* = 4), whereas arrows represent the CLR-transformed loadings of the corresponding microplastic color, shape, or size categories.Analytical subsamples were not entered as independent observations.

Exploratory multivariate analyses were used to visually map the descriptive relationships between MPs morphology and coach class (SL, GL, 3AC, and 1AC) that are not fully apparent from univariate summaries alone. PCA of the CLR-transformed composition matrices showed that the first two principal components explained 94.2% of the variance in color composition, 100.0% in shape composition, and 89.9% in size composition (Figure S2A–C), allowing the PC1–PC2 biplots to summarize the main compositional gradients (Figure 6A–C and Figure S3A–C). PC1 and PC2 explained 59.8% and 34.4% of the variance in the color PCA, and 60.0% and 40.0% of the variance, respectively, in the shape PCA (Figure 6A,B and Figure S3A,B). The CLR transformation accounted for the compositional nature of the percentage data, which convey information only through ratios among categories.

Color loadings showed that PC1 mainly contrasted transparent, red, and yellow-brown particles (positive loadings) with blue and green particles (negative loadings), whereas PC2 was driven mainly by black particles, with secondary contributions from red and yellow/brown (Figure 6A and Figure S3A). 1AC scored high on PC1 and moderately positive on PC2, consistent with a color signature dominated by transparent and warm-colored particles. GL scored strongly negative on PC1 and positive on PC2, consistent with enrichment in black and cooler colors (Figure 6A and Figure S3A). SL and 3AC occupied intermediate positions, indicating more balanced color profiles.

Shape-based PCA showed a simpler structure, driven by the balance between fragments and conglomerates on PC1 and by fibers on PC2 (Figure 6B and Figure S3B). GL and SL scored positively on PC1, indicating a higher relative contribution from fragments, whereas 3AC and 1AC scored negatively, indicating that conglomerates were more influential (Figure 6B and Figure S3B). On PC2, 1AC scored the highest and was associated with fiber loadings, while 3AC scored the lowest. This indicates that 1AC was most strongly associated with a fiber-rich composition, GL and SL were more fragment-associated, and 3AC showed a stronger conglomerate-related signature.

Size-class PCA showed that the larger size fractions (200–500, 500–1000, and 1000–3000 µm) contributed most to PC1, while PC2 was driven by the smallest (<50 µm) and 100–200 µm classes (Figure 6C and Figure S3C). 1AC projected towards the large-size end of PC1, consistent with enrichment in the 500–1000 µm and 200–500 µm fractions, while GL projected towards the smaller size classes, with higher relative contributions from <50 µm and 50–100 µm particles (Figure 6C). SL and 3AC again occupied intermediate positions in the size PCA, indicating broader size distributions than the more clearly differentiated 1AC and GL profiles.

Overall, the exploratory PCA results were consistent with the univariate morphology patterns: 1AC was characterized by a fiber-rich, transparent, larger-size particle profile, while GL showed a darker, fragment-rich, finer-particle profile. In contrast, 3AC showed a more distinct position in the shape PCA because of its association with conglomerate-related composition. Supporting CLR heatmaps are provided in the Supplementary Information (Figure S4).

### 2.4. Microplastics Polymer Identification by µ-Raman Spectroscopy

Micro-Raman spectroscopy was used to confirm polymer identity in the Raman-characterized subset of visually identified suspected MPs. Among successfully characterized particles at 785 nm excitation, PET/polyester accounted for >95% of accepted spectra, supported by spectral matching (HQI ≥ 90%) and diagnostic bands near ~1615 and ~1725 cm^−1^ (Figure S5), ruling out misidentification of biogenic cellulose as a synthetic polymer [41]. This PET-dominant profile aligns with global indoor MPs studies, where PET often accounts for 50–85% of polymers due to its prevalence in textiles, packaging, and building materials [7,42]. Given µ-Raman’s high specificity for PET fibers, this dominance likely reflects an environmental pattern rather than analytical bias [43,44]. The remaining particles included non-plastics like silica, dust, and carbon; others were excluded due to poor signal, fluorescence, or unclear matches. No coach-specific variations in PET prevalence emerged within the analyzed subset.

Most indoor studies describe diverse polymer mixtures, with PET prominent and accompanied by 10–30% shares of polyolefins (PE, PP), polyamides, and other polymers [4,5,24]. In contrast, the present train data reveal a strikingly uniform profile: PET is overwhelmingly dominant, and PE, PP, PVC, and PS were not detected within the Raman-characterized subset. These polymers are commonly associated with non-textile indoor plastic sources such as packaging films and containers (PE, PP), rigid consumer goods and foams (PP, PS), and building or furnishing materials, including vinyl-based components (PVC). This near-exclusive PET dominance differs from the polymer diversity typically reported in other indoor microenvironments, consistent with the idea that indoor MPs assemblages can carry material-specific “fingerprints” tied to material inventories and usage patterns, rather than converging toward a universal, complex mixture [4].

PET, primarily in the form of polyester fibers, is widely used in upholstery, textiles, luggage, bedding, and carpeting in train coaches. High passenger traffic, with constant movement, friction, and abrasion, can be efficiently sheds these fibers [45], whereas less fibrous polymers like PE and PP may be generated or persist less readily in dust [42,46].

PET food packaging and bottles may act as potential secondary sources via fragmentation, crushing, handling, and friction in a commuting setting, which can plausibly generate PET fragments that accumulate in dust, likely yielding irregular fragments rather than fibers. Distinguishing these requires morphology analysis (Section 2.2.2), and indoor evidence points to fiber dominance linked to textiles [5,7,46]. The Trains’ extensive textile surfaces and rapid occupancy amplify this. Polyester-based textiles are well-established sources of microfiber emissions under realistic use scenarios [47].

Comparative data support this interpretation. Homes and offices show PET concentrations of 20–50%, mixed with PP and PE from varied sources [7,48]. Cars can reach 70% PET from upholstery but include road-derived polyolefins. Outdoor air favors PP and PE [46], and a study of hospital dust from the same city reported PET and PE dominance driven by single-use plastics and textiles [9]. Compared with other transit microenvironments, subways report multi-polymer profiles including PET [32].

In these train coaches, the polymer profiles appear distinct, which is hypothesized to be influenced by standardized polyester materials, enclosed coaches that limit external inputs, high mechanical stress that promotes fiber release, and rapid occupancy turnover that enhances textile shedding. Such factors collectively amplify PET while diluting the relative contribution of other polymers. In this train, the >95% PET fraction is among the most uniform polymer profiles reported for indoor MPs environments.

### 2.5. Relative Microplastic Load and Compositional Diversity

This study used comparative and ecological indices to characterize relative MPs accumulation and compositional diversity across train coaches. Because public transport environments lack a pristine, uncontaminated background state, absolute contamination indices were considered inappropriate; instead, the RLR was used to express the fold difference in MPs abundance relative to the GL coach. GL was selected as an internal operational comparator, on account of its lowest measured abundance in this sampling campaign, and was not treated as a background or uncontaminated reference. The RLR values are therefore best read as within-train enrichment factors rather than pollution-classification indices. MPs abundance followed the order GL ˂ 3AC ˂ SL ˂ 1AC, with RLR values of 1.26, 1.93, and 4.06 for 3AC, SL, and 1AC, respectively (Table S7), indicating roughly four times more MPs per gram of settled dust in 1AC than in GL under the sampled conditions.

Compositional diversity, assessed using Simpson’s diversity index across shape, size, and color, followed a pattern consistent with the abundance data: GL showed the most compositionally mixed profile, while 1AC showed the lowest diversity across all three categories despite carrying the highest MPs abundance (Table S8). This combination reflects 1AC’s dominance by fibers, transparent particles, and the 500–1000 µm size fraction. High-abundance, low-diversity assemblages of this kind are typical of confined, textile-rich indoor spaces, where repeated abrasion of furnishings can produce a more homogeneous fibrous MPs profile [6,37], and where limited ventilation and cleaning practices may further favor fiber redistribution and accumulation [5].

Together, the RLR and diversity results point to an accumulation of differences linked to the specific coach enclosure and interior design, consistent with coach- or zone-specific accumulation patterns reported elsewhere [49,50]. Polymer diversity followed a similar pattern of reduced heterogeneity: PET dominance within the Raman-characterized subset contrasts with the more varied polymer mixtures reported in many indoor dust studies [49,50], and likely reflects textile sources, though analytical constraints may have obscured minor polymers, a limitation future ATR-FTIR or expanded Raman screening should address.

Overall, MPs accumulation levels in these train coaches were comparable to those in other crowded indoor environments such as classrooms [50], and the high, compositionally uniform particle loads in 1AC point to a need for targeted material substitution and coach-specific mitigation strategies [51].

### 2.6. Estimated Trip and Annual Microplastic Intake

Exposure-relevant MPs intake through settled-dust ingestion was estimated as trip intake (${EDI}_{Trip}$) and annual intake (EAI) across coach classes, age groups, travel durations, and travel-frequency scenarios, to compare relative particle-intake potential rather than to derive toxicological or health-risk evidence. Because settled dust represents deposited rather than airborne particles, estimates were restricted to inadvertent ingestion; inhalation was not quantified. Station-to-station travel durations were used instead of a single full-route assumption, offering a more granular approximation of exposure for both short- and long-distance passengers, broadly in line with indoor-dust studies linking exposure duration to time spent in a contaminated microenvironment [7].

Deterministic ${EDI}_{Trip}$ increased with travel duration, coach-specific MPs abundance, and age-specific dust ingestion rates. Across scenarios, ${EDI}_{Trip}$ ranged from 5.78 particles/trip (adults/teenagers, GL, shortest route) to 388.35 particles/trip (toddlers, 1AC, full route). For full-route travel, adult ${EDI}_{Trip}$ rose from 31.90 particles/trip in GL to 129.45 in 1AC, and toddler estimates rose from 95.71 to 388.35 particles/trip (Table S9a). The higher toddler values follow directly from their larger assumed ingestion rates and are broadly consistent with evidence that young children face greater dust-related exposure due to hand-to-mouth behavior [52].

Annual intake was driven mainly by travel frequency. Occupational EAI for staff ranged from 1388.00 to 31,068.11 particles/year (Table S9b); passenger EAI spanned 11.57–20,194.27 particles/year across frequency (Table S9a), duration, age, and coach-class scenarios. These estimates indicate that frequent travel can raise cumulative particle intake, especially in higher-MP coaches, but they remain exposure-relevant intake estimates rather than indicators of health outcomes, consistent with recent indoor-dust studies noting that particle-intake metrics support scenario comparison while dose–response evidence for environmental MPs remains limited [24,49].

Monte Carlo simulations across all coach classes, age groups, and travel scenarios reproduced the same coach-wise ordering as the deterministic estimates (GL < 3AC < SL < 1AC; Table S10a,b). Spearman sensitivity analysis identified travel duration, MP concentration, and ingestion rate as the main contributors to passenger ${EDI}_{Trip}$, whereas passenger EAI was most sensitive to travel frequency, then duration and MP concentration (Table S11). For staff, ${EDI}_{Trip}$ and EAI variability were driven mainly by travel duration and MP concentration; ingestion rate contributed less, since only adult staff were modeled.

Together, these results indicate that both microenvironmental MP load and travel behavior shape estimated intake, though the relative contribution of each remains uncertain without empirical validation. Pairing settled-dust sampling with in-transit airborne MP measurements and chemical characterization of particle-associated additives would help clarify the exposure relevance of railway microenvironments [50].

## 3. Limitations and Directions for Future Research

This study provides baseline evidence of MPs contamination in settled dust from a long-distance passenger train. However, the spatial and temporal scope of sampling was restricted to a single train; a single composite dust sample was collected from each of the four coach types, along one route, and during a single-season campaign. Dust was collected post-journey from a limited number of coach types, which does not fully capture day-to-day variability, passenger turnover effects, cleaning-cycle differences, or seasonal changes in ventilation and outdoor infiltration.

Furthermore, because dust was collected without a defined sampling area or controlled accumulation period, the data represent the concentration of MPs within the dust matrix (MPs/g), which can be influenced by dilution from non-plastic environmental dust. Future studies should employ defined area-sampling protocols over known intervals to calculate actual MPs deposition rates (MPs/m^2^/day).

Future multi-route and multi-season studies encompassing various operational cycles are necessary to quantify temporal variability and isolate the specific influences of coach design, occupancy, and cleaning frequency. This study evaluates the matrix concentration of MPs (MPs per gram of settled dust) to estimate inadvertent ingestion exposure, rather than evaluating surface deposition fluxes (MPs/m^2^).

Methodologically, visual identification performed with optical microscopy is standard but constrained by detection limits, which may lead to the underestimation of particles ~ <20 μm. μ-Raman analysis is further limited by analytical limitations. Fluorescence from organic residues and polymer dyes can obscure spectral peaks; although effectively mitigated in this study using a 785 nm laser, the required higher power increases the risk of localized thermal degradation (burning) in pigmented or weathered particles. Additionally, environmental degradation and persistent surface contamination can alter spectral baselines, complicating automated library matching, while the practical identification of extremely fine particles (~1 µm) remains limited by weak signal-to-noise ratios and background interference from the filter substrate. Future research should incorporate routine recovery tests and complementary techniques, such as SEM-EDX or pyrolysis-GC/MS, to improve the resolution of finer fractions and mitigate spectral exclusions caused by fluorescence.

Finally, exposure estimates relied on standard ingestion rates, serving as screening-level comparative indicators rather than definitive toxicological doses. Because this study focused on settled dust, a sink matrix inherently biased toward larger, deposited particles, it primarily reflects inadvertent ingestion and cannot directly resolve real-time airborne dynamics. To build upon this assessment, subsequent research must pair settled-dust inventories with active airborne sampling and size-resolved inhalation dosimetry during travel. Advancing from this baseline toward actionable mitigation will ultimately require intervention-oriented studies, specifically targeting material substitution for high-shedding textiles, and the implementation of enhanced, coach-specific vacuuming and cleaning protocols to effectively manage deposited dust.

## 4. Conclusions

This descriptive case study detected MPs in settled dust composites collected from four coaches of a long-distance passenger train. Coach-level concentrations ranged from 925 to 3755 MPs/g. The observed differences in MP accumulation (1AC > SL > 3AC > GL) may reflect differences in coach design, ventilation, passenger density, luggage volume, and the presence of interior textiles, which could influence MP retention. Fibers dominated the assemblage (~72%), transparent particles were the largest color fraction, and the 500–1000 µm size class was the most common among the visually detected MPs, together pointing to a textile- and furnishing-derived signature shaped by coach-specific settling dynamics.

Micro-Raman spectroscopy confirmed PET dominance (>95%), suggesting a material “fingerprint” tied to standardized furnishings and high-use abrasion rather than a diverse indoor polymer mixture. Relative Load Ratios showed a 4-fold MP enrichment in 1AC over the naturally ventilated baseline coach, consistent with enclosed, textile-rich interiors acting as particle sinks, a pattern that parallels other transit systems [32,35]. This finding positions enclosed transport microenvironments as candidate settled-dust reservoirs worth testing across routes and seasons. Simpson’s diversity metrics reinforced this contrast: GL showed a more compositionally mixed profile, while 1AC combined high abundance with low diversity, dominated by fibers and transparent particles.

$\;{EDI}_{Trip}$ ranged from 5.78 to 388.35 particles/trip, with staff annual intake reaching 31,068.11 particles/year. In the absence of harmonized exposure benchmarks for environmental MPs, these values are best read comparatively across coach types (1AC > SL > 3AC > GL) rather than as absolute risk indicators; Monte Carlo analysis identified dust concentration as the dominant source of uncertainty.

Together, these results identify enclosed, textile-rich coach interiors as potential high-load microenvironments within the sampled train and support targeted mitigation through material selection, maintenance, and ventilation/filtration upgrades and expanded multi-route, multi-season monitoring to refine estimates of variability and inform evidence-based transit health guidance.

## Figures and Tables

**Table 1:** Abundance of microplastics (MPs/g) in settled dust samples from the overall passenger train environment, including total cumulative abundance, mean ± SD, median, range, and coefficient of variation (CV).

Characteristics	Category	Aggregate Sum (MPs/g)	%	Mean	SD	Med	Min	Max	CV
Total		7633	100	1908	1283.28	1477	925	3755	0.67
Color	Red	1056	13.83	264	130.25	226	153	452	0.49
	Blue	781	10.23	195	151.98	161	50	409	0.78
	Green	153	2.00	38	25.54	50	0	53	0.67
	Black	977	12.80	244	196.76	200	77	501	0.81
	Yellow/Brown	74	0.97	19	32.50	4	0	67	1.76
	Transparent	4592	60.16	1148	1069.60	786	318	2702	0.93
Shape	Fiber	5529	72.44	1382	1308.69	901	422	3305	0.95
	Fragment	1821	23.86	455	165.17	460	250	651	0.36
	Conglomerate	283	3.71	71	90.60	42	0	200	1.28
Size	<50 (μm)	547	7.44	137	89.60	130	38	250	0.66
	50–100 (μm)	466	6.34	117	80.48	88	56	234	0.69
	100–200 (μm)	649	8.83	162	19.72	162	141	184	0.12
	200–500 (μm)	1709	23.25	427	316.12	277	254	901	0.74
	500–1000 (μm)	3021	41.10	755	806.64	439	192	1952	1.07
	1000–3000 (μm)	958	13.03	240	116.51	203	143	409	0.49

## Data Availability

The original contributions presented in this study are included in the article/Supplementary Materials. Further inquiries can be directed to the corresponding author.

## Supplementary Materials

The following supporting information can be downloaded at: https://www.mdpi.com/article/10.3390/toxics14090822/s1. Table S1: Summary of sampling metadata and coach microenvironmental conditions for settled dust collected from individual coaches on a long-distance passenger train; Table S2: Coach-specific MP concentration used in exposure estimation; Table S3: Dust ingestion rates; Table S4: Travel duration and frequency scenarios; Table S5: Input matrices and preprocessing for exploratory CLR-PCA; Table S6: Morphological distribution of MPs, reported as absolute counts and fractional percentages, in compartmentalized microenvironments of a railway passenger carriage; Table S7: Mean microplastic concentrations (MPs/g) and Relative Load Ratios (RLR) across the four analyzed railway coach classes. The General (GL) coach serves strictly as an internal operational comparator to quantify the relative fold-enrichment of microplastics; Table S8: Simpson’s diversity index (1 − D) for MP shape, size, and color composition in railway coach dust; Table S9a: Deterministic estimated trip intake and annual intake. ${EDI}_{Trip}$ range represents the shortest-duration to full-route scenarios. The passenger EAI range represents the lowest to highest passenger travel-frequency scenarios across all evaluated durations; Table S9b: ${EDI}_{Trip}$ and EAI for staff; Table S10a: Monte Carlo uncertainty summary for full-route ${EDI}_{Trip}$ uncertainty for adult and toddler passengers; Table S10b: Monte Carlo uncertainty summary for full-route EAI uncertainty for weekly toddler passengers and adult staff; Table S11. Global Spearman rank sensitivity summary for estimated trip intake and annual intake. Figure S1: Representative photographs of Indian Railways coach interiors: (A) a first-class air-conditioned (1AC) compartment with red-themed interior; (B) a three-tier air-conditioned (3AC) compartment with blue-themed interior; Figure S2: Scree plots for CLR-PCA of microplastic morphology. (A) Color, (B) shape, and (C) size scree plots showing eigenvalues and percentage of variance explained by each principal component. PC1 and PC2 together explained 94.2%, 100.0%, and 89.9% of the variance in color, shape, and size composition, respectively, supporting use of the two-dimensional biplots as exploratory compositional visualizations; Figure S3: Variable loadings for CLR-PCA of microplastic morphology. (A) Color, (B) shape, and (C) size contribution plots indicating the relative influence of each morphology category on PC1 and PC2. These loadings identify the dominant color classes, shapes, and size fractions contributing to the observed compositional gradients among coach classes; Figure S4: CLR heatmaps and hierarchical clustering of microplastics morphology. (A) Color CLR heatmap with Euclidean–Ward clustering, showing coach-class-specificenrichment of transparent and dark particles. (B) Shape CLR heatmap distinguishing fiber-rich from fragment-rich environments. (C) Size-class CLR heatmap separating coach classes dominated by intermediate/large versus fine MPs. Colors indicate relative enrichment (red) or depletion (blue) of morphology categories around the CLR-based geometric mean. Figure S5: Representative µ-Raman spectra confirming polyethylene terephthalate (PET) in microplastic particles. Spectra are displayed after baseline correction and normalization [53,54,55].

## Abbreviations

The following abbreviations are used in this manuscript:

1AC	First-class sleeper air-conditioned coach.
3AC	Three-tier sleeper air-conditioned coach.
AC	Air-conditioned/air conditioning.
ATR-FTIR	Attenuated total reflection Fourier-transform infrared spectroscopy.
CLR	Centered log-ratio.
CV	Coefficient of variation.
EAI	Estimated annual intake.
EDI_trip_	Estimated daily intake per trip.
GL	General unreserved coach.
HQI	Hit quality index.
MPs	Microplastics.
P05/P50/P95	5th/50th (median)/95th percentiles.
PCA	Principal component analysis.
PE	Polyethylene.
PET	Polyethylene terephthalate.
PP	Polypropylene.
PS	Polystyrene.
PVC	Polyvinyl chloride.
Pyrolysis-GC/MS	Pyrolysis-gas chromatography/mass spectrometry.
QA/QC	Quality assurance/quality control.
RLR	Relative load ratio.
SD	Standard deviation.
SDI	Simpson’s diversity index.
SEM-EDX	Scanning electron microscopy with energy dispersive X-ray spectroscopy.
SI	Supplementary Information.
SL	Reserved non-air-conditioned sleeper coach.
