# Supplementary material for: Microplastics in Settled Dust from Indian Long-Distance Railway Coaches: Baseline Occurrence, Relative Load, and Scenario-Based Ingestion Estimates
Source: Toxics. 2026 Sep 15;14(9):822. doi: 10.3390/toxics14090822 (PMC13610999; doi:10.3390/toxics14090822)
Supplement: Supplementary file 1 [file toxics-14-00822-s001.zip › toxics-4517716-supplementary.pdf]

Microplastics in Settled Dust from Indian Long-Distance Railway Coaches: Baseline Occurrence, Relative Load, and Scenario-Based Ingestion Estimates

Authors' Information:

Nisarg Mehta[1], Jing Wang[2,3*], Barbara Kozielska[1]

[1]Silesian University of Technology, Faculty of Energy and Environmental Engineering, Department of Air Protection, 22B Konarskiego St., 44-100 Gliwice, Poland.

[2]Empa, Laboratory for Building Energy Materials and Components, Überlandstrasse 129, 8600 Dübendorf, Switzerland.

[3]ETH Zürich, Institute of Environmental Engineering, Laura-Hezner-Weg 7, 8093 Zürich, Switzerland.

[*]Corresponding Author

Corresponding Author:

Prof. Jing Wang

Phone: +41 44 633 36 21

E-mail: jing.wang@ifu.baug.ethz.ch

Abbreviations

1AC — first-class air-conditioned coach.

3AC — three-tier air-conditioned coach.

ATR–FTIR — attenuated total reflectance–Fourier-transform infrared spectroscopy.

CLR — centered log-ratio.

EAI — estimated annual intake.

EDI_trip — estimated trip intake.

FTIR — Fourier-transform infrared spectroscopy.

GL — general unreserved coach.

HQI — hit quality index.

IR — dust ingestion rate.

MP — microplastic.

MPs — microplastics.

OM — organic matter.

P05 — 5th percentile.

P50 — 50th percentile or median.

P95 — 95th percentile.

PC1 — first principal component.

PC2 — second principal component.

PCA — principal component analysis.

PE — polyethylene.

PET — polyethylene terephthalate.

PP — polypropylene.

PS — polystyrene.

QA/QC — quality assurance and quality control.

RLR — relative load ratio.

SL — reserved non-air-conditioned sleeper coach.

TF — annual travel frequency.

UV–VIS–NIR — ultraviolet–visible–near-infrared.

Symbols used in equations

C_i— MP concentration in settled dust from coach class i, particles/g.

C_{GL}— MP concentration in the GL coach, particles/g.

D_s— travel duration under scenario s, h.

IR_a— dust ingestion rate for age group a, g/day.

n_i— particle count in morphology category i.

N— total count across categories included in a diversity calculation.

k— number of morphology categories.

RLR_i— relative load ratio for coach class i.

TF— annual travel frequency, trips/year.

Table S1. Summary of sampling metadata and coach microenvironmental conditions for settled dust collected from individual coaches on a long-distance passenger train.

Coach Type	Route Duration (h)	Last cleaning before sampling	AC / Ventilation Status	Window & Door Status During Travel	Textiles Present (Upholstery/Curtains/Bedding)	Doors	Windows	Passenger Area	Passenger occupancy
**GL**	27.6	13-17 hr	Non-AC (Natural)	Open	Vinyl, or artificial leatherette/No curtains/No bedding	6	36	≈ 58–60 m^2 (≈ 630–645 sq. ft)	100-108
**SL**			Non-AC (Natural)	Open	Vinyl, or artificial leatherette/No curtains/No bedding	4	36		78
**3AC**			Air-Conditioned	Sealed/Closed	Vinyl, or artificial leatherette/curtains/bedding provided	4	18		72
**1AC**			Air-Conditioned	Sealed/Closed	Vinyl, or artificial leatherette/curtains/bedding provided	4	10		24

Protocol

S1. Sample Pre-Treatment

A validated two-step protocol, adapted with minor modifications from previously established methodologies [1–6], was applied to ensure accurate isolation of MPs and minimize false positives from organic matter. Initially, any large debris was removed from the dust samples by sieving them through a 5 mm metal mesh. The samples were then transferred to glass evaporating dishes and dried at 50°C for 12 hours to remove moisture. Following this, 1 gram of the dried dust from each sample was accurately measured, and transferred into a 100 mL glass beaker.

The next step involved the chemical digestion of organic material (OM) using 30% hydrogen peroxide (Fisher Scientific, Loughborough, UK), to reduce the organic-matter content before density separation. Prior to a ~5-day incubation, samples were sonicated (SONOREX, BANDELIN, Germany) for 15 minutes to enhance digestion efficiency. After the digestion process, the samples were heated on a hotplate (uniSTIRRER 7, LLG labware, Germany) at 50°C to completely evaporate any residual H_2O_2. The resulting digested material was then rinsed three times with deionized water and dried again.

For the density separation, 50 mL of pre-filtered (using a Whatman® GF/C glass-fiber filter; 1.2 µm pore size, 47 mm diameter) zinc chloride ($ZnCl_2$) (Sigma-Aldrich, St. Louis, MO, USA) solution of density 1.6 g cm^{-3} was added to each beaker. The mixture was agitated to ensure all particles were suspended. After letting the particles settle for about 30 minutes, the supernatant was carefully transferred to centrifuge tubes and centrifuged (5430 R, EPPENDORF, Germany) at 4500 rpm for 10 minutes.

The MPs were collected from the supernatant via vacuum filtration, using filter paper (Cytiva, Whatman®, GlobalLife Sciences Solution Operations UK Ltd., Buckinghamshire, UK; mixed cellulose ester grid filter paper; 0.2 µm pore size, 47 mm diameter). To optimize MP recovery, the $ZnCl_2$ extraction step was repeated three times for each sample, employing the same filter across all filtrations. The filters were then air-dried in covered glass Petri dishes at room temperature and stored in cabinets designed to minimize contamination until analysis. This approach ensures robust elimination of organic contaminants and enhanced MP retrieval through multiple density separation cycles, in line with conventional methods for MP examination.

S2 Sample Analysis

S2.1. Morphological analysis and quantification of MPs

The morphological characterization of MPs was performed according to the standardized categorization proposed by Hartmann et al. [7]. Dried filters were observed under a Leica S9i stereomicroscope (Leica Microsystems, Wetzlar, Germany) at 50× magnification, a resolution optimized for discerning sub-millimeter particles while minimizing optical distortion. Quantitative assessments of particle dimensions were conducted via Leica Acquire software (version 3.4.7; Build 9121). MPs were systematically classified by shape (fragments, conglomerates and fibers), color (red, blue, transparent, black, yellow/brown, green) and size (<50 μm, 50–100 μm, 100–200 μm, 200–500 μm, 500–1000 μm, and 1000–3000 μm).

S2.2. Polymer characterization

The polymer composition of MPs was elucidated via micro-Raman spectroscopy employing a LabRAM HR Evolution UV-VIS-NIR spectrometer (HORIBA Scientific, Kyoto, Japan), adhering to the rigorous protocols outlined by Kashfi et al. [1] for reproducible polymer fingerprinting in complex environmental matrices. A near-infrared 785 nm laser at 300 mW power was selected as the excitation source to suppress fluorescence interference, a prevalent artifact in pigmented or degraded MPs, while acquiring spectra across the diagnostically informative 250–2000 cm^{-1} fingerprint region, encompassing key vibrational modes for common polymers such as polyethylene, polypropylene, and polystyrene. Spectral datasets were systematically matched against curated reference libraries (e.g., HORIBA's polymer database) using spectral correlation algorithms, achieving >90% identification confidence thresholds to mitigate false positives inherent to automated matching in heterogeneous samples. Across 12 analytical batches, a total of 110 particles (after the exclusion of particles that exhibited thermal degradation under laser excitation) were analyzed by micro-Raman spectroscopy. This subset represents an analytical fraction of approximately 12% of the total visually quantified microplastics.

S3. Quality assurance/quality control

To minimize contamination from ambient MPs, strict quality-control measures were applied throughout dust sampling, sample preparation, and analysis. Only glass and metal equipment was used; personnel wore nitrile gloves and cotton lab clothing, and all surfaces and instruments were

cleaned with ethanol before each procedure. Reagents were filtered through 1.2 μm glass fiber filters (Whatman® GF/C). During organic matter digestion, beakers and inactive vacuum pumps were covered with aluminum foil to prevent airborne contamination.

Procedural blanks were processed in parallel with samples using identical reagents and protocols (three blanks) to assess background interference. Sample handling and analysis were conducted in a dedicated, low-traffic laboratory with windows closed and air conditioning off. All containers were kept covered when possible. No MPs (>50 μm) were detected in blanks by stereo microscopy, and no accepted synthetic polymer matches attributable to laboratory contamination were obtained from the procedural blanks, indicating background contamination below detection limits. These measures align with established MP quality-control standards and provide a check for potential laboratory contamination.

S4. Exposure-intake calculation and Monte Carlo uncertainty analysis

S4.1. Estimated trip and annual intake

Exposure-relevant MPs intake from settled-dust ingestion was estimated using the estimated trip intake (EDI_{Trip}) and the estimated annual intake (EAI). EDI_{Trip} was calculated as

$$EDI_{Trip} = C_i \times IR_a \times \frac{D_s}{24}$$

where C_i is the MP concentration in settled dust for coach class I, expressed as particles/g dust; IR_a is the age-specific dust ingestion rate, expressed as g/day; and D_s is the travel duration for scenario s, expressed in hours. Because ingestion rates were expressed in g/day, no mg-to-g conversion factor was applied.

$$EAI = EDI_{Trip} \times TF$$

where *TF* is the annual travel frequency, expressed as trips/year.

Age-specific dust ingestion rates were adopted from Liao et al. [8]. The ingestion rates used were 0.055 g/day for infants, 0.090 g/day for toddlers, 0.060 g/day for children, 0.030 g/day for teenagers, and 0.030 g/day for adults.

S4.2. Monte Carlo uncertainty analysis

Monte Carlo simulation was performed to propagate uncertainty in ingestion-based particle-intake estimates. Coach-specific MP concentrations were represented by triangular distributions using the minimum, mean, and maximum replicate concentrations for each coach class (Table S2). Age-specific dust ingestion rates were also represented by triangular distributions, using the Liao et al. [8] values as the mode and ±20% as the lower and upper bounds (Table S3). Travel duration and annual travel frequency were treated as fixed scenario variables (Table S4).

For each coach class, age group, travel-duration scenario, and annual travel-frequency scenario, 20,000 iterations were performed in R using A fixed random seed of 7 for reproducibility. The analysis was conducted using "*dplyr", "tidyr", "purrr",* and *"readr"* packages for data handling, scenario generation, iteration, and output export. Random sampling from triangular distributions was performed using a custom R function, while percentile summaries and Spearman rank correlations were calculated using base R functions. Outputs were summarized as the 5th, 50th, and 95th percentiles (P05, P50, P95) for EDI_{Trip} and EAI.

Spearman rank correlations were calculated as exploratory sensitivity indicators. For EDI_{Trip}, sensitivity was evaluated against MP concentration, dust ingestion rate, and travel duration. For EAI, sensitivity was evaluated against MP concentration, dust ingestion rate, travel duration, and annual travel frequency.

Table S2. Coach-specific MP concentration used in exposure estimation.

Coach	*n*	C_{min}	$C_{mode/mean}$	C_{max}	Unit
GL	3	240	925.33	1602	MPs/g
3AC	3	601	1168.33	2153	MPs/g
SL	3	1302	1785.67	2103	MPs/g
1AC	2	2703	3754.50	4806	MPs/g

Table S3. Dust ingestion rates.

Age group	IR_{min}	IR_{mode}	IR_{max}	Unit
Infant	0.044	0.055	0.066	g/day
Toddler	0.072	0.090	0.108	g/day
Child	0.048	0.060	0.072	g/day
Teenager	0.024	0.030	0.036	g/day
Adult	0.024	0.030	0.036	g/day

IR_{mode} values were adopted from Liao et al [8]. IR_{min} and IR_{max} represent ±20% uncertainty bounds used for triangular Monte Carlo distributions.

Table S4. Travel duration and frequency scenarios.

Scenario type	Values
Travel duration	5, 8.5, 14.5, 23, and 27.583 h/trip
Passenger frequency	2, 12, and 52 trips/year
Staff frequency	240 trips/year

S5. Relative load ratio and compositional diversity.

Because public transport environments lack a pristine, uncontaminated background state, traditional absolute contamination indices were not applicable. Instead, a descriptive Relative Load Ratio (RLR) was calculated to evaluate the internal accumulation gradient across the sampled coach classes. The GL coach, which exhibited the lowest microplastic abundance, was utilized strictly as an internal operational comparator to calculate the fold-difference in microplastic concentrations across the train system, rather than as an environmental background.

The RLR was calculated as:

$$RLR_i = \frac{C_i}{C_{GL}}$$

where RLR_i is the relative load ratio for coach class i, C_i is the MP concentration in coach class i expressed as MPs/g of settled dust, and C_{GL} is the MP concentration measured in the GL coach. The GL coach was selected because it showed the lowest MP abundance in this sampling campaign. It was used strictly as an internal operational comparator and not as an uncontaminated reference or environmental background. Accordingly, RLR values are interpreted only as within-train relative-load ratios and are not used to assign pollution classes or contamination severity.

The Simpson diversity index $(1 - \mathrm{D})$ is calculated as [9,10]:

$$1 - \mathrm{D} = 1 - \sum_{i=0}^{k} \left(\frac{n_i}{N}\right)^2$$

where $\mathrm{n_i}$ is the grid-extrapolated particle count in category i, N is the total grid-extrapolated count across all categories included in the corresponding morphology block, and *k* is the number of categories. Higher values indicate a more compositionally diverse and even distribution, whereas lower values indicate stronger dominance by one or a few categories.

S6. Exploratory compositional analysis

Three separate compositional matrices were prepared for color, shape, and size. There are three shape categories (fiber, fragment, conglomerate), six size classes (<50, 50–100, 100–200, 200–500, 500–1000, and 1000–3000 µm), and six color categories (red, blue, green, black, yellow-brown, and transparent).

To remove the influence of unequal total particle abundance among samples, the raw count matrix was converted to row-wise percentages. For each sample, the count for each morphological category was divided by the total count across all categories and multiplied by 100, yielding a relative-abundance matrix suitable for compositional analysis. Because the resulting variables represent parts of a whole and therefore are constrained by constant-sum dependence, the percentage data were transformed using the centered log-ratio (CLR) method. Before CLR transformation, a pseudocount equal to half of the smallest non-zero percentage value in the dataset was added to all entries to avoid undefined logarithmic values in categories containing zeros, allowing all variables to be retained while respecting the compositional structure of the dataset.

PCA was performed on the CLR-transformed matrix to summarize the principal axes of variation in microplastic morphology and to visualize relationships among samples and morphological variables in reduced-dimensional space. For interpretative clarity in the biplots, variables were grouped into three predefined classes—shape, size, and color—although the PCA was conducted on the full CLR-transformed variable matrix.

For heatmap-based clustering, the CLR-transformed data were analyzed using Euclidean distances and Ward's minimum-variance algorithm (ward.D2) for both samples and variables. Clustered heatmaps were generated from the CLR matrix using a diverging blue–white–red color scale. In this representation, negative CLR values indicate relative depletion and positive values indicate relative enrichment of a given morphological feature relative to the sample-wise geometric mean. Variables were grouped into three major morphological categories (shape, size, and color) to facilitate interpretation of clustered patterns.

All analyses and figure generation were performed in R using the packages compositions*, "FactoMineR", "factoextra","pheatmap","grid"*, and related "*tidyverse*" tools. Output files included the raw count matrix, row-wise percentage matrix, CLR-transformed matrix, PCA results, clustered heatmaps, and associated distance matrices for reproducibility and downstream verification.

Table S5: Input matrices and preprocessing for exploratory CLR-PCA.

	SL	GL	3AC	1AC
**Color**				
Red	11.99	16.54	20.29	12.04
Blue	22.91	16.43	14.55	1.33
Green	2.80	0	4.54	1.33
Black	4.31	31.89	8.9	13.34
Yellow/Brown	3.75	0.76	0	0
Transparent	54.23	34.38	51.71	71.96
**Shape**				
Fiber	59.83	45.62	62.84	88.02
Fragment	36.47	52.54	37.16	6.66
Conglomerates	3.70	1.84	0	5.33
**Size**				
Size_lt_50	9.08	11.34	3.25	7.03
Size_50_100	13.61	8.26	4.79	2.84
Size_100_200	10.70	15.53	14.81	4.25
Size_200_500	15.53	27.97	24.57	25.34
Size_500_1000	27.28	21.15	34.93	54.91
Size_1000_3000	23.79	15.75	17.64	5.63

Table S6. Morphological distribution of MPs, reported as absolute counts and fractional percentages, in compartmentalized microenvironments of a railway passenger carriage.

		Coach class							
**Characteristics**	**Category**	**SL**		**GL**		**3AC**		**1AC**	
		**n**	**%**	**n**	**%**	**n**	**%**	**n**	**%**
**Total**		**1785**	**100**	**925**	**100**	**1168**	**100**	**3755**	**100**
**Color**	Red	214	11.99	153	16.54	237	20.29	452	12.04
	Blue	409	22.91	152	16.43	170	14.55	50	1.33
	Green	50	2.80	0	0.00	53	4.54	50	1.33
	Black	77	4.31	295	31.89	104	8.90	501	13.34
	Yellow/Brown	67	3.75	7	0.76	0	0.00	0	0.00
	Transparent	968	54.23	318	34.38	604	51.71	2702	71.96
**Shape**	Fiber	1068	59.83	422	45.62	734	62.84	3305	88.02
	Fragment	651	36.47	486	52.54	434	37.16	250	6.66
	Conglomerates	66	3.70	17	1.84	0	0.00	200	5.33
**Size**	<50 (μm)	156	9.08	103	11.34	38	3	250	7.03
	50-100 (μm)	234	13.61	75	8.26	56	5	101	2.84
	100-200 (μm)	184	10.70	141	15.53	173	15	151	4.25
	200-500 (μm)	267	15.53	254	27.97	287	25	901	25.34
	500-1000 (μm)	469	27.28	192	21.15	408	35	1952	54.91
	1000-3000 (μm)	409	23.79	143	15.75	206	18	200	5.63

Size classification, only particles with measurable dimensions were included; conglomerates were excluded from the size denominator..

Table S7. Mean microplastic concentrations (MPs/g) and Relative Load Ratios (RLR) across the four analyzed railway coach classes. The General (GL) coach serves strictly as an internal operational comparator to quantify the relative fold-enrichment of microplastics.

Coach class	MPs concentration (MPs/g)	RLR based on GL	Interpretation
GL	925.33	1.00	Internal comparator
3AC	1168.33	1.26	1.26-fold higher than GL
SL	1785.67	1.93	1.93-fold higher than GL
1AC	3755.50	4.06	4.06-fold higher than GL

Table S8. Simpson's diversity index (1 − D) for MP shape, size, and color composition in railway coach dust.

Coach	Shape	Size	Color
GL	0.515	0.808	0.726
3AC	0.467	0.761	0.660
SL	0.508	0.807	0.635
1AC	0.218	0.624	0.450

Table S9a. Deterministic estimated trip intake and annual intake. EDI_{Trip} range represents the shortest-duration to full-route scenarios. The passenger EAI range represents the lowest to highest passenger travel-frequency scenarios across all evaluated durations.

Coach	Age group	Full-route EDI_{Trip} (particles/trip)	Passenger EDI_{Trip} range (particles/trip)	Passenger EAI range (particles/year)
GL	Adult/Teenager	31.90	5.78–31.90	11.57–1659.03
GL	Infant	58.49	10.60–58.49	21.21–3041.55
GL	Child	63.81	11.57–63.81	23.13–3318.05
GL	Toddler	95.71	17.35–95.71	34.70–4977.08
3AC	Adult/Teenager	40.28	7.30–40.28	14.60–2094.70

3AC	Infant	73.85	13.39–73.85	26.77–3840.28
3AC	Child	80.57	14.60–80.57	29.21–4189.40
3AC	Toddler	120.85	21.91–120.85	43.81–6284.10
SL	Adult/Teenager	61.57	11.16–61.57	22.32–3201.51
SL	Infant	112.87	20.46–112.87	40.92–5869.44
SL	Child	123.14	22.32–123.14	44.64–6403.03
SL	Toddler	184.70	33.48–184.70	66.96–9604.54
1AC	Adult/Teenager	129.45	23.47–129.45	46.93–6731.42
1AC	Infant	237.33	43.02–237.33	86.04–12340.94
1AC	Child	258.90	46.93–258.90	93.86–13462.85
1AC	Toddler	388.35	70.40–388.35	140.79–20194.27

Table S9b. $\mathrm{EDI}_{\mathrm{Trip}}$ and EAI for staff.

**Coach**	**Staff $\mathrm{EDI}_{\mathrm{Trip}}$ range (particles/trip)**	**Staff EAI range (particles/year)**
GL	5.78–31.90	1388.00–7657.04
3AC	7.30–40.28	1752.50–9667.84
SL	11.16–61.57	2678.50–14776.21
1AC	23.47–129.45	5631.75–31068.11

Table S10a. Monte Carlo uncertainty summary for full-route EDI_{Trip} uncertainty for adult and toddler passengers.

**Coach**	**Adult P05–P50–P95**	**Toddler P05–P50–P95**	**Unit**
GL	15.35–31.59–48.75	46.44–94.89–146.33	particles/trip
3AC	27.45–44.06–66.29	82.45–132.81–198.28	particles/trip
SL	47.61–59.54–72.20	141.97–178.22–216.08	particles/trip

Coach	Adult P05–P50–P95	Toddler P05–P50–P95	Unit
1AC	100.32–128.80–160.76	301.80–386.42–481.95	particles/trip

Table S10b. Monte Carlo uncertainty summary for full-route EAI uncertainty for weekly toddler passengers and adult staff.

Coach	Weekly toddler passenger P05–P50–P95	Adult staff P05–P50–P95	Unit
GL	2424–4942–7591	3704–7576–11610	particles/year
3AC	4255–6866–10326	6546–10569–15791	particles/year
SL	7425–9293–11254	11410–14302–17378	particles/year
1AC	15643–20126–25062	24225–30938–38609	particles/year

Table S11. Global Spearman rank sensitivity summary for estimated trip intake and annual intake.

**Exposure group**	**Output**	**Input variable**	**Spearman ρ**
Passenger	EDI_{Trip}	Travel duration	0.653
Passenger	EDI_{Trip}	MP concentration	0.574
Passenger	EDI_{Trip}	Dust ingestion rate	0.432
Passenger	EAI	Travel frequency	0.823
Passenger	EAI	Travel duration	0.362
Passenger	EAI	MP concentration	0.324
Passenger	EAI	Dust ingestion rate	0.245
Staff	EDI_{Trip}/EAI	Travel duration	0.731
Staff	EDI_{Trip}/EAI	MP concentration	0.639
Staff	EDI_{Trip}/EAI	Dust ingestion rate	0.092

(A) (B)

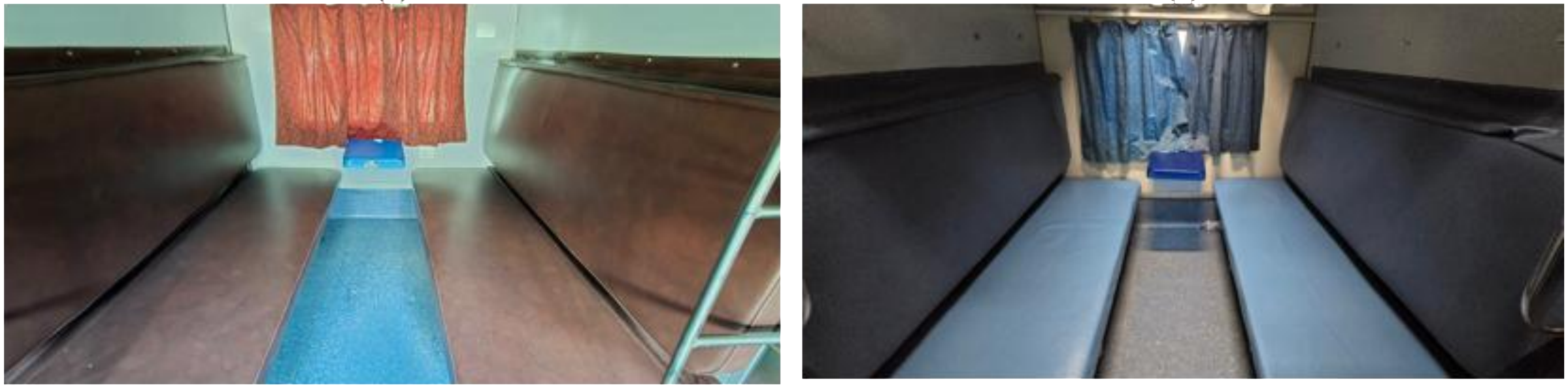

Figure S1. Representative photographs of Indian Railways coach interiors: (A) a first-class air-conditioned (1AC) compartment with red-themed interior; (B) a three-tier air-conditioned (3AC) compartment with blue-themed interior.

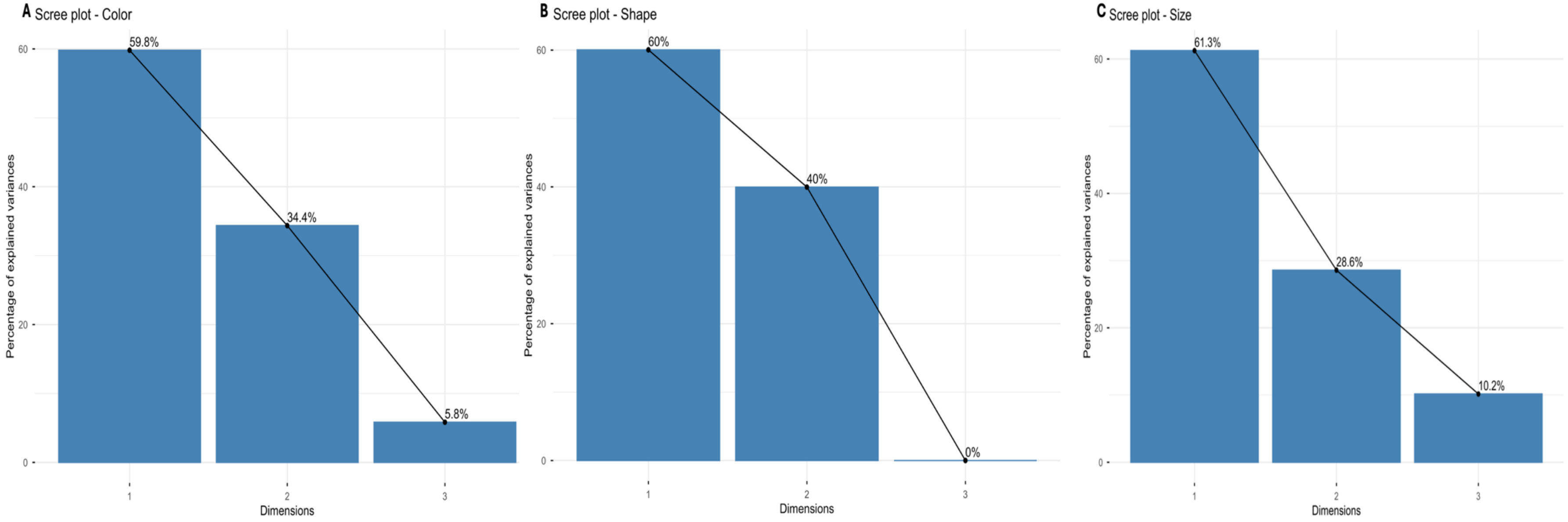

Figure S2. Scree plots for CLR-PCA of microplastic morphology. (A) Color, (B) shape, and (C) size scree plots showing eigenvalues and percentage of variance explained by each principal component. PC1 and PC2 together explained 94.2%, 100.0%, and 89.9% of the variance in color, shape, and size composition, respectively, supporting use of the two-dimensional biplots as exploratory compositional visualizations.

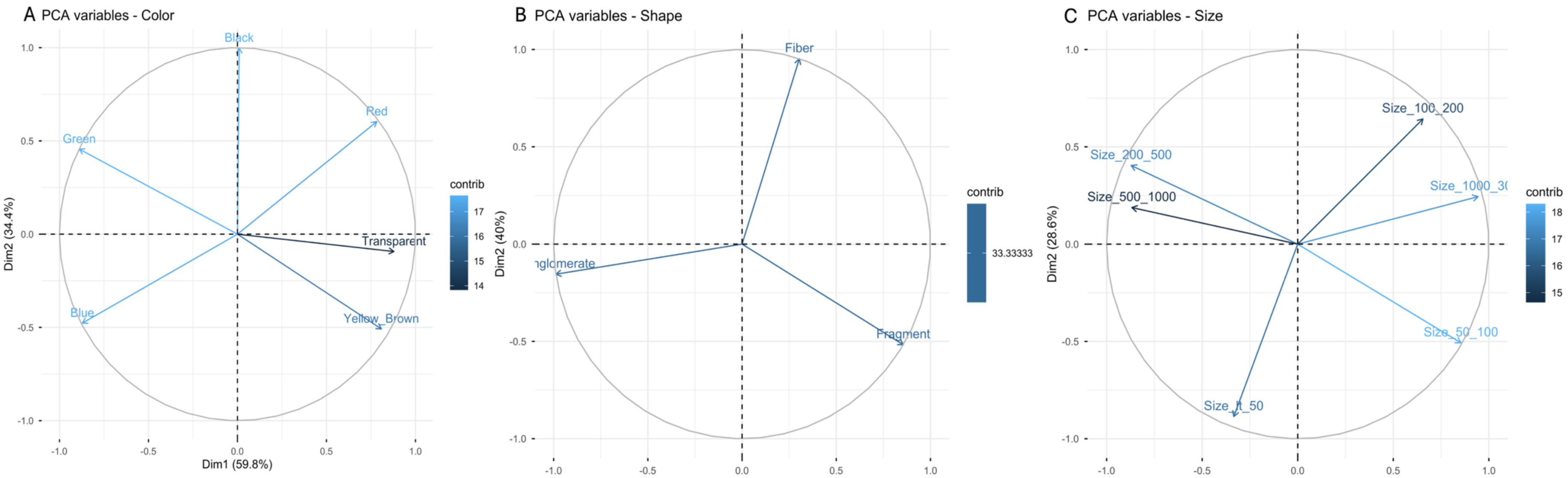

Figure S3. Variable loadings for CLR-PCA of microplastic morphology. (A) Color, (B) shape, and (C) size contribution plots indicating the relative influence of each morphology category on PC1 and PC2. These loadings identify the dominant color classes, shapes, and size fractions contributing to the observed compositional gradients among coach classes.

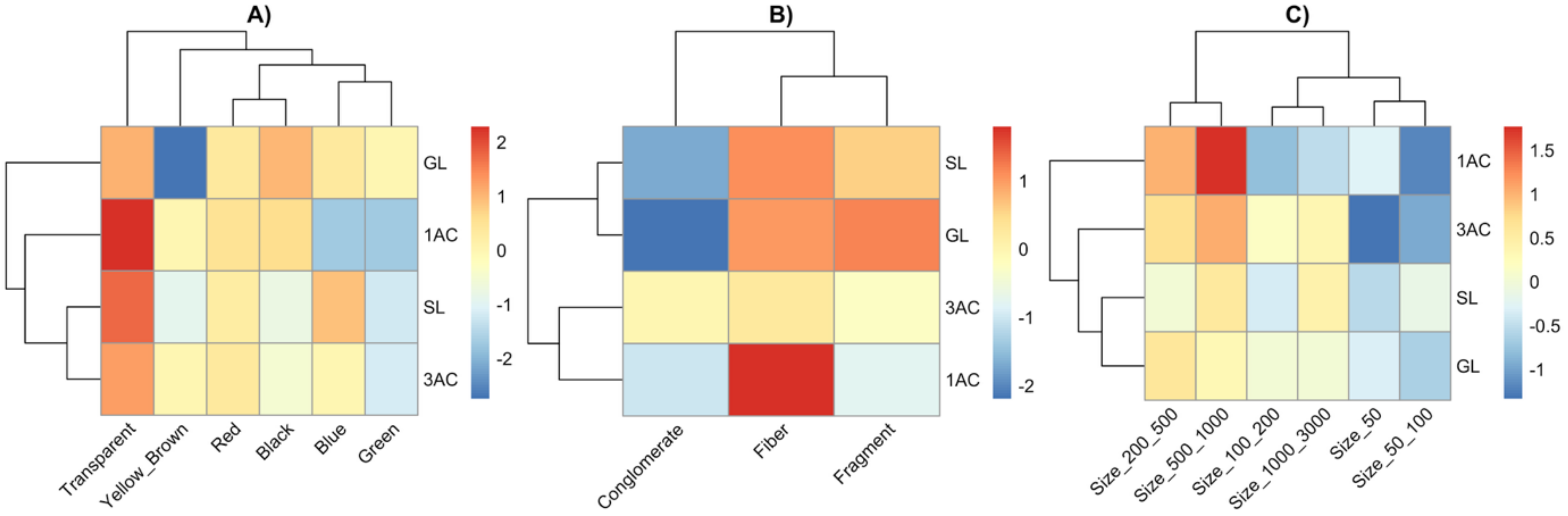

Figure S4. CLR heatmaps and hierarchical clustering of microplastics morphology. (A) Color CLR heatmap with Euclidean–Ward clustering, showing coach-class-specificenrichment of transparent and dark particles. (B) Shape CLR heatmap distinguishing fiber-rich from fragment-rich environments. (C) Size-class CLR heatmap separating coach classes dominated by intermediate/large versus fine MPs. Colors indicate relative enrichment (red) or depletion (blue) of morphology categories around the CLR-based geometric mean.

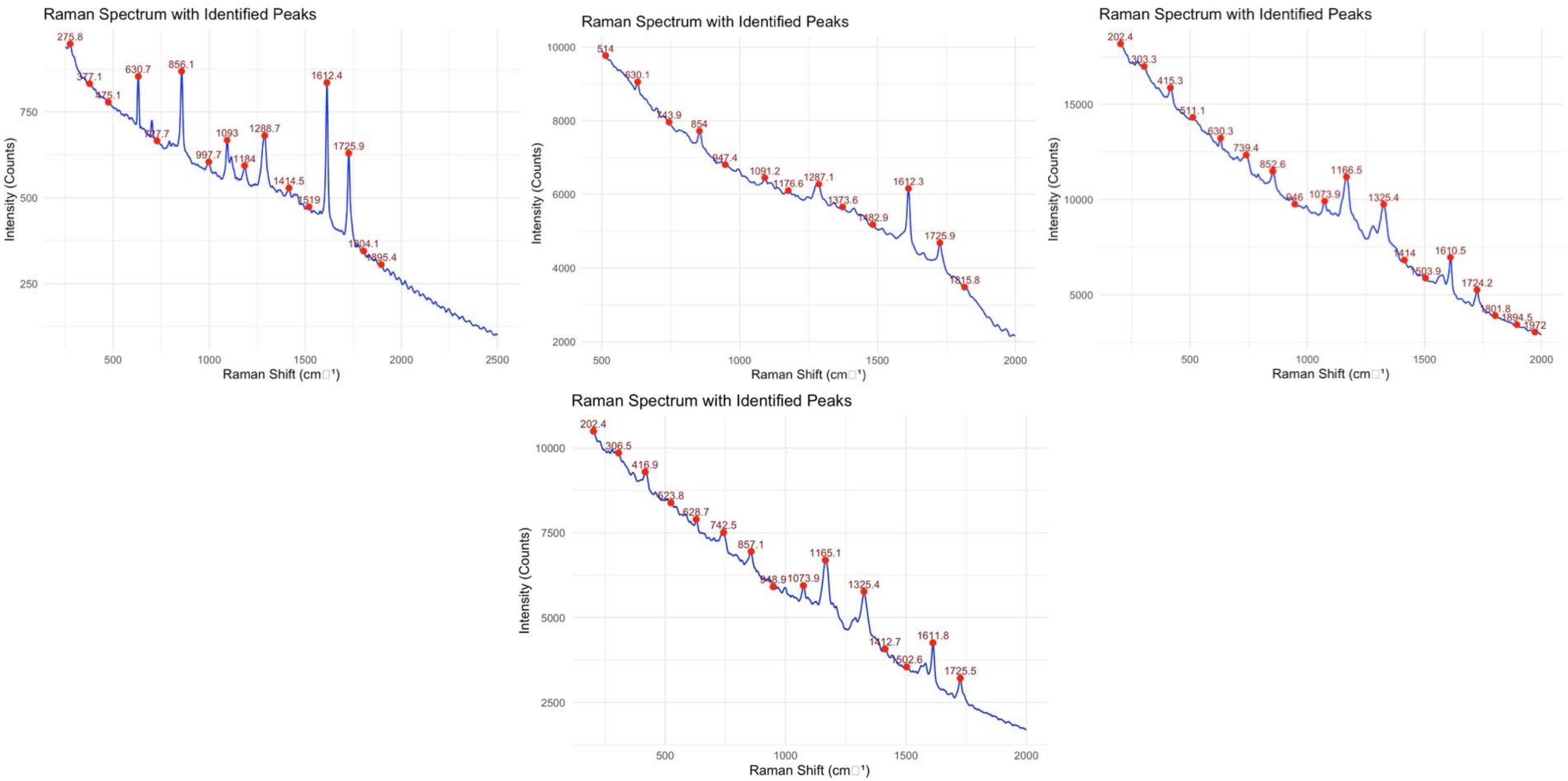

Figure S5. Representative μ-Raman spectra confirming polyethylene terephthalate (PET) in microplastic particles. Spectra are displayed after baseline correction and normalization.

References:

1. Kashfi, F.S.; Ramavandi, B.; Arfaeinia, H.; Mohammadi, A.; Saeedi, R.; De-la-Torre, G.E.; Dobaradaran, S. Occurrence and exposure assessment of microplastics in indoor dusts of buildings with different applications in Bushehr and Shiraz cities, Iran. *Sci. Total Environ.* **2022**, *829*, 154651. https://doi.org/10.1016/j.scitotenv.2022.154651.
2. Mehta, N.; Kozielska, B.; Lunagaria, J.; Ladva, K.D. Characterization and exposure assessment of indoor microplastics in hospital indoor settled dust: First insides from India. *Build. Environ.* **2025**, *279*, 113065. https://doi.org/10.1016/j.buildenv.2025.113065.
3. Mehta, N.; Wang, J.; Kozielska, B. Assessment of Indoor Microplastics in Settled Dust at a High-Traffic Transit Building: Characterization, Abundance, Polymer Profiling, and Ingestion Exposure. *J. Environ. Sci.* **2026**, S1001074226002767. https://doi.org/10.1016/j.jes.2026.04.007.
4. Mehta, N.; Kozielska, B. A systematic literature review and bibliometric analysis of sources and analytical approaches of indoor microplastics. *Emerg. Contam.* **2024**, *10*, 100274. https://doi.org/10.1016/j.emcon.2023.100274.
5. Mehta, N.; Kozielska, B. Disposable Plasticware Production as a Source of Microplastics in Settled Dust: Quantification, Characterization, and Exposure Assessment. *Atmosphere* **2026**, *17*, 282. https://doi.org/10.3390/atmos17030282.
6. Zhu, J.; Zhang, X.; Liao, K.; Wu, P.; Jin, H. Microplastics in dust from different indoor environments. *Sci. Total Environ.* **2022**, *833*, 155256. https://doi.org/10.1016/j.scitotenv.2022.155256.
7. Hartmann, N.B.; Hüffer, T.; Thompson, R.C.; Hassellöv, M.; Verschoor, A.; Daugaard, A.E.; Rist, S.; Karlsson, T.; Brennholt, N.; Cole, M.; Herrling, M.P.; Hess, M.C.; Ivleva, N.P.; Lusher, A.L.; Wagner, M. Are We Speaking the Same Language? Recommendations for a Definition and Categorization Framework for Plastic Debris. *Environ. Sci. Technol.* **2019**, *53*, 1039–1047. https://doi.org/10.1021/acs.est.8b05297.
8. Liao, C.; Liu, F.; Guo, Y.; Moon, H.-B.; Nakata, H.; Wu, Q.; Kannan, K. Occurrence of Eight Bisphenol Analogues in Indoor Dust from the United States and Several Asian Countries: Implications for Human Exposure. *Environ. Sci. Technol.* **2012**, *46*, 9138–9145. https://doi.org/10.1021/es302004w.
9. Wang, T.; Zou, X.; Li, B.; Yao, Y.; Zang, Z.; Li, Y.; Yu, W.; Wang, W. Preliminary study of the source apportionment and diversity of microplastics: Taking floating microplastics in the South China Sea as an example. *Environ. Pollut.* **2019**, *245*, 965–974. https://doi.org/10.1016/j.envpol.2018.10.110.
10. SIMPSON, E. Measurement of Diversity. Nature 163, 688 (1949). https://doi.org/10.1038/163688a0
